# Anisotropic ferroelectric distortion effects on the RKKY interaction in topological crystalline insulators

**DOI:** 10.1038/s41598-021-84398-0

**Published:** 2021-03-05

**Authors:** Hosein Cheraghchi, Mohsen Yarmohammadi

**Affiliations:** 1 School of Physics, Damghan University, 36716-41167 Damghan, Iran; 2 School of Physics, Institute for Research in Fundamental Sciences (IPM), 19395-5531 Tehran, Iran; 3 Department of Energy Engineering and Physics, Amirkabir University of Technology, 14588 Tehran, Iran; 4 Lehrstuhl für Theoretische Physik I, Technische Universität Dortmund, Otto-Hahn Straße 4, 44221 Dortmund, Germany

**Keywords:** Applied physics, Condensed-matter physics, Quantum physics

## Abstract

Manipulation of electronic and magnetic properties of topological materials is a topic of much interest in spintronic and valleytronic applications. Perturbation tuning of multiple Dirac cones on the (001) surface of topological crystalline insulators (TCIs) is also a related topic of growing interest. Here we show the numerical evidence for the ferroelectric structural distortion effects on the Ruderman-Kittel-Kasuya-Yosida (RKKY) interaction between two magnetic impurity moments on the SnTe (001) and related alloys. The mirror symmetry breaking between Dirac cones induced by the ferroelectric distortion could be divided into various possible configurations including the isotropically gapped, *coexistence of gapless and gapped*, and anisotropically gapped phases. Based on the retarded perturbed Green’s functions of the generalized gapped Dirac model, we numerically find the RKKY response for each phase. The distortion-induced symmetry breaking constitutes complex and interesting magnetic responses between magnetic moments compared to the pristine TCIs. In the specific case of coexisted gapless and gapped phases, a *nontrivial* behavior of the RKKY interaction is observed, which has not been seen in other Dirac materials up until now. For two impurities resided on the same sublattices, depending on the distortion strength, magnetic orders above of a critical impurity separation exhibit irregular ferromagnetic ⇔ antiferromagnetic phase transitions. However, independent of the impurity separation and distortion strength, no phase transition emerges for two impurities resided on different sublattices. This essential study sheds light on magnetic properties of Dirac materials with anisotropic mass terms and also makes TCIs applications relatively easy to understand.

## Introduction

Exploring and discovering topological materials have become intriguing due to their exotic transport properties^1,2^. In three-dimensional topological insulators (TIs), topology is protected by the time-reversal invariant surface state which crosses the Fermi level, residing in the bulk band gap^3,4^. All strong TIs are robust against all crystal terminations, possessing an odd number of the Dirac cones in their surface Brillouin zone (SBZ)^2,5^. Though crystalline symmetries are less robust against crystal deformations, their variety and abundance could lead to copious new kinds of surface Dirac cones. In 2011, Liang Fu found that the time-reversal symmetries can be replaced with the crystal symmetries to support nontrivial topological phases and to protect gapless surface electronic states^6^. This perspective has been confirmed by the discovery of topological crystalline insulators (TCIs) with the topology protected by symmorphic^7,8^ and nonsymmorphic^9,10^ crystal symmetries. The first experimentally realization of TCIs belong to the (001) surface of lead thin salt family in IV-VI semiconductors, namely SnTe, $\hbox {Pb}_{1-x}\hbox {Sn}_x$Se ($x > 0.2$) and $\hbox {Pb}_{1-x}\hbox {Sn}_x$Te ($x > 0.4$)^7,8,11^, in which two interacting coaxial Dirac cones dubbed a double surface Dirac cone.

It is worthwhile noting that in addition to TCIs comprising of multiple surface Dirac cones, there are many topological materials introducing such states, for instance, LaBi as a compound of the family of rare-earth monopnictides with a strongest spin–orbit coupling is identified as a time-reversal invariant TI associated with three Dirac cones in its surface band structure, two coexist at the corners and one at the center of the Brillouin zone^12^. Moreover, multiple Dirac cones hidden in Bismuth high-$T_{\mathrm{c}}$ superconductors introducing candidates for both electron- and hole-doped topological insulators have theoretically been reported employing the first-principle calculations^13^. In another work, multiple Dirac cones have been found in honeycomb-monolayer transition metal trichalcogenides using the *ab initio* calculations to show the nontrivial topological magnetism in these systems^14^, which can be tuned by tensile strain. Very recently, three Dirac cones at the Brillouin zone center of $\hbox {MnBi}_4\hbox {Te}_7$ and $\hbox {MnBi}_6\hbox {Te}_{{10}}$ have been found both theoretically and experimentally^15^. On the Dirac nature of charge carriers in graphene, heterostructutre of graphene layers which are separated by 2D polar insulating systems, can be treated as an effectively spinless and intrinsically magnetic TCI possessing multiple Dirac surface states^16^. In addition to these works, one may find many works from various research groups in this regard. All these works highlight that the physical properties of topological materials possessing multiple surface Dirac cones are remarkable and fascinating.

Semiconductor and metallic spintronics have been attracted great attention in the last decades, using the spin states of the electron as a (quantum) bit of information to encode the data^17,18^. However, the spin-spin exchange interaction is of importance couplings in quantum physics, tuning the magnetic order of the material. The perturbative interaction between two localized magnetic impurities well-known in condensed-matter physics is described by the Ruderman-Kittel-Kasuya-Yosida (RKKY) theory^19–21^, which is tightly connected to the spin-spin correlation function. Recently, various magnetic ordering originating from different mechanisms has been reported in topological materials. Ferromagnetic ordering has been investigated in magnetically doped semiconductors through the mechanism of van Vleck paramagnetism^22,23^. Furthermore, the magnetic texture effects on the spin susceptibilities in Weyl and Dirac semimetals have been studied^24–26^. It has been found that the spin-momentum locking and the internode process in Weyl semimetals play a significant role in spin susceptibility components^27,28^. The effect of Rashba splitting on the spin susceptibility of the undoped and doped TI thin films have been addressed^29^. Overall, the RKKY interaction in topological and Dirac systems is of most importance phenomenon due to showcasing tunable long-range spin-spin couplings^30–34^.

Tunability of topological surface states due to the presence of four valleys on the (001) surface of IV-VI semiconductors make TCIs good candidates for spintronic and valleytronic applications^8,11,35–37^, inducing interesting physics. To some extent, tuning the spin and valley degrees of freedom improves the performance of materials, for example, for non-dissipative and low-energy devices. This, in turn, leads to a topic of much interest; the manipulation of the RKKY interaction with the aid of valley tuning. This manipulation can be done using different methods. Among the extensive body of theoretical studies, physical perturbations are of the straight ways to manipulate the valleys and eventually the RKKY interaction in TCIs. Particularly, the rich interplay between the crystal symmetry and the electronic structure in TCIs can be affected in the presence of perturbations, resulting in the symmetry breaking and the band gap opening in the Dirac cones^7,38–40^.

For this purpose, we will focus on the ferroelectric structural distortion, which historically has provided an interesting anisotropic symmetry breaking and gapped Dirac cones^7,40^. The band gap opening on the (001) surface of TCIs allows us to tune the spin-spin correlation function when two separated magnetic moments reside on the surface. Regarding the presence of the four Dirac cones on TCI’s surface, by the ferroelectric structural distortion, it is easily achievable to provide the coexistence of the gapless and gapped Dirac cones or iso- and aniso-tropically gapped phases. So, the question is how the RKKY interaction, which is closely coupled to the energy dispersion relation, can be influenced by this symmetry breaking, especially at the above-mentioned phases. Owing to the presence of such interesting symmetry breaking, the RKKY interaction in gapped TCIs is expected to present different behaviors compared to the pristine TCIs.

The aforementioned distortion is time-reversal invariant but breaks rotational symmetry, leading to a ferroelectric phase and eventually to the anisotropic Dirac mass terms^7^. Thereby, the metallic surface states acquire a direction-dependent band gap. On the other hand, due to the direct relation between the gapped and/or gapless states and RKKY coupling, it is worth exploring the new physics happening in the presence of these anisotropically gapped states. Regarding the anisotropy in the gaps, we believe that this work explains different physics compared to the pristine TCI. Pristine TCI possesses an isotropic band spectrum in which the interference terms mostly control RKKY couplings. However, in the present work, employing imaginary frequency representation, we investigate the interplay between anisotropy in gaps and interference terms to determine RKKY response. Moreover, the topology of the band spectrum might be changed during the variation in the sign and size of the four gaps leading to a trace for topological phase transitions in RKKY coupling. Our results, especially on the coexistence of the effects of the gapless and gapped phases on the spin-spin correlation function between magnetic impurities on the TCI’s surface have not been reported to date and we claim that such a unique feature may increase interests in TCIs for further electrical and magnetical properties. It is expected that RKKY coupling decreases with the gap value, however, in the case of coexisted gapless and gapped phases, quite different results are expected to be observed, since on the one hand, massless Dirac fermions are responsible for the charge carriers and on the other hand, massive ones play a role, introducing a new quasi-fermion in the entire system. These, in turn, manifest themselves in the RKKY responses, as presented in the paper.

In this case, analytical derivation of the RKKY interaction is very difficult, hence, to calculate the perturbed RKKY coupling, firstly, we have implemented the analytical retarded Green’s function obtained from the perturbed low-energy Hamiltonian of TCIs and secondly, the RKKY interaction has numerically been calculated. We find that the resulting RKKY coupling reflects the surface isotropic/anisotropic band gap opening, leading to the magnetic phase transition when two magnetic moments reside on the *same* sublattices. However, for the magnetic moments on *different* sublattices, the RKKY interaction cannot show a different magnetic ordering. The resultant physics can provide useful insights to tailor magnetic ordering in TCIs. What would be the advance of this work on RKKY in Dirac materials is the presence of multiple gapped Dirac cones in TCI distributed on four points in SBZ. Here RKKY interaction is calculated in some interesting topological phases with different gap sizes and signs. Moreover, the anisotropy of the band spectrum results in non-trivial behaviors in RKKY interaction. One of them is the enhancement of RKKY interaction with the gap size during passing a critical impurity separation which is not trivial. We would state that the presence of mirror symmetry concerning the (110) plane in SnTe and related alloys have been experimentally confirmed in the angle-resolved photoemission spectroscopy (ARPES) experiments^8,11,35^ and we believe that on the experimental side, the predicted results for the surface states of SnTe in the presence of anisotropic ferroelectric distortion in the present work can be readily detected in ARPES and tunneling spectroscopy experiments.

The rest of the paper is organized as follows. In "Theoretical background" section, we first introduce the theoretical background of pristine TCIs. Then we present the electronic features of TCIs in the presence of ferroelectric distortion. In "Low-energy RKKY coupling in TCIs in the presence of ferroelectric distortion" section, we calculate the RKKY interaction in gapped TCIs and show the numerical results when two magnetic impurities are on the same and different sublattices. Finally, the paper ends in "Summary" section with a summary of the remarkable findings.

**Figure 1:**
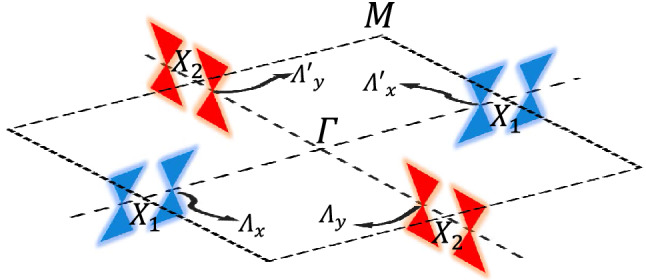
Surface Brillouin zone of pristine SnTe (001) plane. Four Dirac cones at {$\Lambda _x$ and $\Lambda '_x$} near the $X_1$ point and {$\Lambda _y$ and $\Lambda '_y$} near the $X_2$ point of SBZ form the surface states in TCIs.

## Theoretical background

### Electronic features in unperturbed TCIs: from four-band to two-band model

We begin by representing the reported angle-resolved-photoemission-spectroscopy experiments^8,11,35^ of the surface states in (001) plane of SnTe and related alloys such as $\hbox {Pb}_{1-x}\hbox {Sn}_x$Te and $\hbox {Pb}_{1-x}\hbox {Sn}_x$Se. The “unperturbed” effective Hamiltonians of four Dirac cones at {$\Lambda _x$ and $\Lambda '_x$} near the $X_1$ point and {$\Lambda _y$ and $\Lambda '_y$} near the $X_2$ point of SBZ forming surface states in TCIs are given by [we set $\hbar = 1$ for simplicity throughout the present work]

1a $$\begin{aligned} {\mathscr {H}}_{X_1}(\mathbf{k }) = {}&\left[ \eta _1 k_x \sigma _y - \eta _2 k_y \sigma _x \right] \otimes \tau _0 + n \tau _x + \delta \sigma _y \tau _y\, , \end{aligned}$$

1b $$\begin{aligned} {\mathscr {H}}_{X_2}(\mathbf{k }) = {}&\left[ \eta _2 k_x \sigma _y - \eta _1 k_y \sigma _x \right] \otimes \tau _0 + n \tau _x + \delta \sigma _x \tau _y\, , \end{aligned}$$

where the three terms describe respectively two copies of Dirac cones, the term which shifts the energy of these two Dirac cones to form two new high-energy Dirac cones with opposite energies and the term which shifts two copies of Dirac cones to have opposite chiralities. The Pauli matrices $\vec {\sigma } = (\sigma _0,\sigma _x,\sigma _y,\sigma _z)$ and $\vec {\tau } = (\tau _0,\tau _x,\tau _y,\tau _z)$ respectively act in the spin and space. Researches were first dealt only with the first term, while the atomically sharp interface between the system and the vacuum results in two additional term, so-called intervalley scattering terms^39^. Although extra momentum $\mathbf{k }=(k_x,k_y)$-dependent terms have been added to these models later, they do not affect the experimentally observed physics of TCIs significantly^41,42^. The used parameters in this paper $\eta _1 = 3.53$ eV.Å , $\eta _2 = 1.91$ eV.Å , $n = 0.055$ eV, and $\delta = 0.04$ eV are taken from Refs.^38,40^.

**Figure 2:**
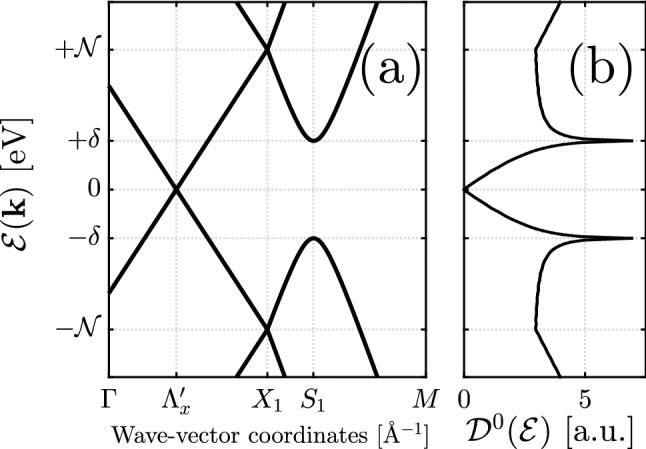
(**a**) Energy spectrum of Dirac fermions in the vicinity of the $X_{1}$ point along the lines $\Gamma -X_1$ and $X_1-M$ consisting of Dirac point $\Lambda '_x$ and saddle point $S_1$. The density of surface states (in arbitrary units) of unperturbed TCIs shows two van Hove singularities at energies $\pm \delta$ corresponding to the saddle points, see panel (**b**).

TCI (001) plane of SnTe or related alloys possesses ${\mathscr {C}}_4$ rotation symmetries along the *x* and *y* directions, implying that one may focus on $X_1$ only and use the relation $X_2 = {\mathscr {C}}_4 X_1 {\mathscr {C}}^{-1}_4$ to capture the physics around the $X_2$ point. Of other symmetries, ${\mathscr {C}}_2$ is important as well between Dirac cones themselves so that $\Lambda '_x = {\mathscr {C}}_2 \Lambda _x {\mathscr {C}}^{-1}_2$ and $\Lambda '_y = {\mathscr {C}}_2 \Lambda _y {\mathscr {C}}^{-1}_2$. Of course, we also allow to conclude $\Lambda _y = {\mathscr {C}}_4 \Lambda _x {\mathscr {C}}^{-1}_4$ and $\Lambda '_y = {\mathscr {C}}_4 \Lambda '_x {\mathscr {C}}^{-1}_4$^38–40^. Figure 1 captures the configurations of four Dirac cones in the SBZ of (001) plane in TCIs. The symmetry breaking perturbations do not enter Eq.(1) and will be introduced at the level of low-energy two-band model.

Focusing the above points, the energy spectrum of surface states in the vicinity of $X_1$ point from Eq. (1a) is given by

2 $$\begin{aligned} {\mathscr {E}}^\mu _{X_1}(\mathbf{k })={}\mu \sqrt{{\mathscr {N}}^2 + \eta ^2_1 k^2_x +\eta ^2_2 k^2_y \pm 2 \sqrt{{\mathscr {N}}^2 \eta ^2_1 k^2_x + n^2 \eta ^2_2 k^2_y}}, \end{aligned}$$

where ${\mathscr {N}}^2 = n^2 + \delta ^2$, $\mu = +(-)$ refers to the conduction (valence) band and $+(-)$ inside the square root stands for the upper (lower) conduction and lower (upper) valence band. We print the band structure along the lines $\Gamma -X_1$ and $X_1-M$ in the SBZ [see Fig. 1] in Fig.2a. The system is gapless at Dirac point $\Lambda '_x = (-{\mathscr {N}}/\eta _1,0)$, while the gap opens at saddle point $S_1 = (0,+n/\eta _2)$. We stress that using the rotational symmetries ${\mathscr {C}}_2$ and ${\mathscr {C}}_4$, one simply obtains other Dirac and saddle points.

A physical quantity particularly useful for such dense systems is the density of states (DOS) in which the electronic correlations between charge carriers is translated to the Green’s functions using the identity

3 $$\begin{aligned} G^0(\mathbf{k },{\mathscr {E}})=\frac{1}{{\mathscr {E}}+\texttt {i} o^{+}-{\mathscr {H}}_{X_1}(\mathbf{k })}\, , \end{aligned}$$

where $o^+ = 5$ meV is a very small real number. This Green’s function is related to the DOS via

4 $$\begin{aligned} {\mathscr {D}}^0({\mathscr {E}})=-\frac{1}{\pi }\sum _{\mathbf{k } \in \mathrm {SBZ}} \mathrm {Im}\, \left[ \mathrm {Tr}\, G^{0}(\mathbf{k },{\mathscr {E}})\right] \, , \end{aligned}$$

The specific nature of Green’s function elements will be the subject of the RKKY interaction of perturbed TCIs in "Low-energy RKKY coupling in TCIs in the presence of ferroelectric distortion" section. Having the diagonal elements of the Green’s function matrix, one obtains DOS, as shown in Fig. 2b. From Eq. (3), DOS diverges at singularities of the numerator, occurring at energies $\pm \delta$ corresponding to the saddle points, leading to emerged van Hove singularities. These singularities remind a Lifshitz transition [a deformation of the Fermi surface]^38,40^. This procedure will be altered when the system is perturbed.

Here we adopt one simplification with a view for applying and/or calculating the ferroelectric distortion-induced RKKY coupling in TCIs. We immediately turn to the two-band model by linearizing the Hamiltonian near the Dirac cones by starting with the $\Lambda _x$ point, since the gapless phase of surface states originates from the Dirac cones. Thus, we leave the saddle points here since it is gapped naturally. We achieve the following low-energy Hamiltonian^43^

5 $$\begin{aligned} {\mathscr {H}}^0_{\Lambda _x} = {\tilde{\eta }}_1 p_x \sigma _y -{\tilde{\eta }}_2 p_y \sigma _x \, , \end{aligned}$$

To this end, $p_x = k_x - \Lambda _x$ measuring from the $\Lambda _x$ point, $p_y = k_y$, ${\tilde{\eta }}_1 = (2{\mathscr {N}}/n)\eta _1$ and ${\tilde{\eta }}_2 = (2\delta /n)\eta _2$ are required to be produced. Eventually, the energy spectrum is given by

6 $$\begin{aligned} {\mathscr {E}}^{0,\mu }_{\Lambda _x}(\vec {p})={}\mu \sqrt{{\tilde{\eta }}^2_1 p^2_x + {\tilde{\eta }}^2_2 p^2_y}\, , \end{aligned}$$

describing the linear dispersion energy of massless fermions along the line $\Gamma -X_1$ in the (001) plane of TCIs [see left side of Fig. 2b]. We adopt one more simplification here with the aid of definitions $v_{\mathrm{F}} = \sqrt{{\tilde{\eta }}_1 {\tilde{\eta }}_2}$, ${\mathscr {K}}_x = \sqrt{{\tilde{\eta }}_1/{\tilde{\eta }}_2}\,p_x$ and ${\mathscr {K}}_y = \sqrt{{\tilde{\eta }}_2/{\tilde{\eta }}_1}\,p_y$. Hence, we have

7a $$\begin{aligned} {\mathscr {H}}^0_{\Lambda _x} = {}&v_{\mathrm{F}}\left( {\mathscr {K}}_x \sigma _y -{\mathscr {K}}_y \sigma _x \right) \, , \end{aligned}$$

7b $$\begin{aligned} {\mathscr {E}}^{0,\mu }_{\Lambda _x}(\vec {{\mathscr {K}}})={}&\mu \, v_{\mathrm{F}} |\vec {{\mathscr {K}}}|\, . \end{aligned}$$

We will follow Eq. (7a) hereafter as the effective low-energy Hamiltonian, describing the basic physics of gapless surface states. Using the transformations $\{\sigma _i \mapsto -\sigma _i$ and ${\mathscr {K}}_i \mapsto -{\mathscr {K}}_i\}$ [$i \in \{x,y\}$] for two-fold ${\mathscr {C}}_2$ and $\{\sigma _y \mapsto -\sigma _x, \sigma _x \mapsto \sigma _y, {\mathscr {K}}_y \mapsto -{\mathscr {K}}_x, {\mathscr {K}}_x \mapsto {\mathscr {K}}_y\}$ for four-fold ${\mathscr {C}}_4$ rotational symmetries for the pristine TCIs, one may list the Hamiltonian of other Dirac cones in the SBZ as ${\mathscr {H}}^0_{\Lambda '_x} = {\mathscr {H}}^0_{\Lambda _x}$, ${\mathscr {H}}^0_{\Lambda _y} = v_{\mathrm{F}}\left( \tilde{{\mathscr {K}}}_x \sigma _y -\tilde{{\mathscr {K}}}_y \sigma _x \right)$ and ${\mathscr {H}}^0_{\Lambda '_y} = {\mathscr {H}}^0_{\Lambda _y}$, with the definitions of $\tilde{{\mathscr {K}}}_x = ({\tilde{\eta }}_2/{\tilde{\eta }}_1)\,{\mathscr {K}}_x$ and $\tilde{{\mathscr {K}}}_y = ({\tilde{\eta }}_1/{\tilde{\eta }}_2)\,{\mathscr {K}}_y$.

Let us apply the ferroelectric structural distortion which acts as a perturbation in the system.

### Electronic features in TCIs in the presence of ferroelectric distortion

In this part, we first express the two-band Hamiltonian for TCI (001) surface states in the presence of ferroelectric distortion. Because the most essential physics of susceptibility is linked to the DOS, we apply our analysis to establish the ferroelectric distortion-induced electronic features in TCIs.

Two-band Hamiltonian of ferroelectric distortion effects is simply given by a mass term originating from the atomic displacements. This displacement may be produced by strain or electric field, resulting in crystal symmetry breaking and eventually a ferroelectric phase. Derivation of the following Hamiltonian can be tracked in Refs.^7,40^. Although this symmetry breaking leads to the band gap opening at surface Dirac cones, it may be anisotropic and direction-dependent due to the orientation-dependent displacement. From this point, we introduce the corresponding distortion Hamiltonians as

8 $$\begin{aligned} {\mathscr {H}}^{\mathrm{FD}}_{\Lambda _x} = {} +\Delta _{x\mathrm{F}} \tau _z \quad ,\quad {\mathscr {H}}^{\mathrm{FD}}_{\Lambda '_x} = {} -\Delta _{x\mathrm{F}} \tau _z\, ,\quad ,\quad {\mathscr {H}}^{\mathrm{FD}}_{\Lambda _y} = {} +\Delta _{y\mathrm{F}} \tau _z \quad ,\quad {\mathscr {H}}^{\mathrm{FD}}_{\Lambda '_y} = {} -\Delta _{y\mathrm{F}} \tau _z\, . \end{aligned}$$

Adding these terms to the corresponding pristine Hamiltonians, one may find the gapped phase for surface states. Again, we focus on the $\Lambda _x$ here inspiring the presence of two- and four-fold rotational symmetries in pristine TCI. By this, we obtain

9a $$\begin{aligned} {\mathscr {H}}_{\Lambda _x} = {}&\begin{pmatrix} +\Delta _{x\mathrm{F}} &{} v_{\mathrm{F}}\left( -{\mathscr {K}}_y -\mathtt{i} {\mathscr {K}}_x\right) \\ \\ v_{\mathrm{F}}\left( -{\mathscr {K}}_y + \mathtt{i} {\mathscr {K}}_x\right) &{} -\Delta _{x\mathrm{F}} \end{pmatrix}\, , \end{aligned}$$

9b $$\begin{aligned} {\mathscr {E}}^\mu _{\Lambda _x}(\vec {{\mathscr {K}}})={}&\mu \sqrt{v^2_{\mathrm{F}} {\mathscr {K}}^2 + \Delta ^2_{x\mathrm{F}}}\, . \end{aligned}$$

Using the aforementioned ${\mathscr {C}}_2$ and ${\mathscr {C}}_4$ rotational symmetries, the Hamiltonian of other gapped Dirac cones in the SBZ can be listed as^36,40^

10a $$\begin{aligned} {\mathscr {H}}_{\Lambda '_x} = {}&\begin{pmatrix} -\Delta _{x\mathrm{F}} &{} v_{\mathrm{F}}\left( -{\mathscr {K}}_y -\mathtt{i} {\mathscr {K}}_x\right) \\ \\ v_{\mathrm{F}}\left( -{\mathscr {K}}_y + \mathtt{i} {\mathscr {K}}_x\right) &{} +\Delta _{x\mathrm{F}} \end{pmatrix}\, , \end{aligned}$$

10b $$\begin{aligned} {\mathscr {H}}_{\Lambda _y} = {}&\begin{pmatrix} +\Delta _{y\mathrm{F}} &{} v_{\mathrm{F}}\left( -\tilde{{\mathscr {K}}}_y -\mathtt{i} \tilde{{\mathscr {K}}}_x\right) \\ \\ v_{\mathrm{F}}\left( -\tilde{{\mathscr {K}}}_y + \mathtt{i} \tilde{{\mathscr {K}}}_x\right) &{} -\Delta _{y\mathrm{F}} \end{pmatrix}\,, \end{aligned}$$

10c $$\begin{aligned} {\mathscr {H}}_{\Lambda '_y} = {}&\begin{pmatrix} -\Delta _{y\mathrm{F}} &{} v_{\mathrm{F}}\left( -\tilde{{\mathscr {K}}}_y -\mathtt{i} \tilde{{\mathscr {K}}}_x\right) \\ \\ v_{\mathrm{F}}\left( -\tilde{{\mathscr {K}}}_y + \mathtt{i} \tilde{{\mathscr {K}}}_x\right) &{} +\Delta _{y\mathrm{F}} \end{pmatrix}\,. \end{aligned}$$

**Figure 3:**
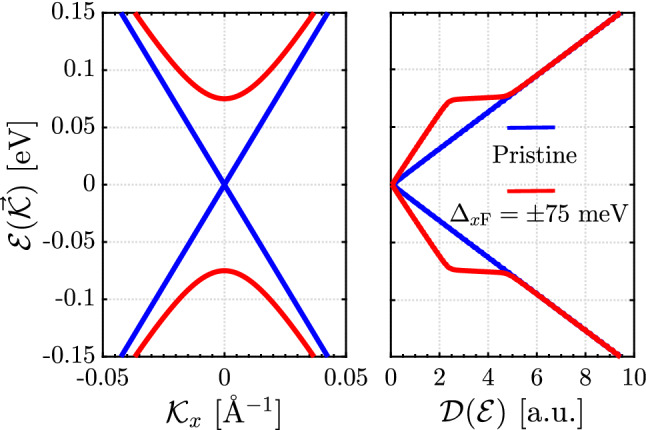
Two-band energy spectrum (left panel) and DOS of TCIs (right panel) in the absence and presence of an arbitrary ferroelectric gap $\Delta _{x\mathrm{F}} = \pm 75$ meV. The distortion is applied along one direction only making two Dirac cones gapped and keeps two rest ones gapless in the SBZ. The total DOS of all these gapless and gapped Dirac cones are plotted in the right panel.

By evaluating Eq. (9) with the mass term $\Delta _{x\mathrm{F}}$, one obtains the gapped dispersing bands at Dirac cones, as represented in Fig. 3 [the bands appear similarly with different slopes if one plots ${\mathscr {E}}(\vec {{\mathscr {K}}})$ as a function of ${\mathscr {K}}_y$]. One, in the left panel of Fig. 3, observes that the bands get apart from each other to open a gap of $2 \Delta _{x\mathrm{F}}$ keeping the Fermi energy at zero energy. It should be pointed out that for the two-band model, van Hove singularities are absent in DOS due to the linear dispersion energy of Dirac fermions as well as due to throwing away saddle points as the origin of the DOS divergences. As shown in the right panel of Fig. 3, the electronic DOS presents a different trend for surface states. It should be mentioned that we have plotted the total DOS here by summing over the Green’s functions from both $X_1$ and $X_2$ points, i.e. Eq. (4) is rewritten as ${\mathscr {D}}({\mathscr {E}})=-\frac{2}{\pi }\sum _{\mathbf{k } \in \mathrm {SBZ}} \mathrm {Im}\, \left[ \mathrm {Tr}\, G^{0}_{X_1}(\mathbf{k },{\mathscr {E}}) + \mathrm {Tr}\, G^{0}_{X_2}(\mathbf{k },{\mathscr {E}})\right]$, where Eq. (3) can be employed to find the Green’s functions around $X_1$ and $X_2$ points. As can be seen, the final band of the system is gapless, giving rise to the relevance of RKKY compared to the van Vleck mechanism. Out of the gap, despite of a tiny difference close to the band edges, the value of DOS is almost the same for the pristine TCI compared to the special case, coexistence of gapped and gapless Dirac cones. Similarly, one finds the band structure other Dirac cones, however; the gap sizes may be different applying different $\Delta _{x\mathrm{F}}$ and $\Delta _{y\mathrm{F}}$ simultaneously. Simultaneous displacement means that the displacement is not applied purely along the *x*- or *y*-direction. This, in turn, leads to valley-dependent electronic features on the (001) surface of TCIs. Since the distortion does not add/remove particles to/from the system, the number of states should be preserved, meaning that the area under DOS is conserved in the absence and presence of ferroelectric distortion. However, to apply this constraint on DOS, we need to work with full band spectrum of SnTe crystal. In this work, Hamiltonian is derived based on an effective theory at low energies, and without loss of any behavior of susceptibility, we leave this constraint in Fig. 3.

Nevertheless, in the case of gapped TCI, the relevance of RKKY response is questionable by definition since there is no any states inside the gap around the Fermi energy. Let us comment on this point before delving into the RKKY results. Of the other well-known interactions for magnetic coupling is given by the van Vleck mechanism. It is demonstrated that for chemical potentials inside the gap, the van Vleck interaction between magnetic impurities located on the surface of a thin film of topological insulator is very large and relevant which is stemming from the strong spin-orbit coupling observed in $\hbox {Bi}_2\hbox {Te}_3$ family^22^. This large interaction is responsible for strong ferromagnetism observed in TI thin films^22^. The origin of such ferromagnetic ordering comes back to the large coupling matrix elements of $S_z$ between the conduction and valence band states which is sizable in the band inverted phase. It is also demonstrated that the van Vleck interaction is controlled by the structural inversion asymmetry^44^. However, two-band effective Hamiltonian represented in Eq. (7a) is written based on the *p*-orbitals of Sn and Te atoms^40^ without any mixing. Moreover, the gap opened here is originated from the breaking of mirror symmetry induced by the ferroelectric distortion when spin-orbit coupling is ruled out at low energies. As a result, the van Vleck interaction in gapped TCIs is quite small. In such situation, although RKKY coupling is quite small too, RKKY interaction is a relevant quantity. Indeed, as shown in the integration of Eq. (13), valence band states are involved to the response of magnetic impurities even if the Fermi level lies inside the gap. So the value of susceptibility would be very smaller than the case in which the Fermi level falls inside the conduction band. Based on a self-consistent calculation on the surface spectrum of TIs, the structure of RKKY interaction is preserved in the presence of the gap induced by exchange field of magnetic impurities, nevertheless, its strength is considerably suppressed^45^. It should be mentioned that the RKKY interaction is also calculated inside the gap energies of TI induced by proximity of a superconductor on top of TI surface^46^. Furthermore, RKKY interaction is relevant in the presence of local gaps induced by doped magnetic impurities on the surface of TI^47^. We will explain the fingerprints of the gapped Dirac ones in our system in RKKY coupling in the next section.

## Low-energy RKKY coupling in TCIs in the presence of ferroelectric distortion

To obtain the coupling ${\mathscr {J}}$ between two magnetic moments with the separation of $\mathbf{R } = \mathbf{R }_2 - \mathbf{R }_1$ considering different lattice sites $\{\alpha ,\beta \} = 1,2$, one allows to use the second-order perturbation theory resulting in

11 $$\begin{aligned} J(\mathbf{R }) = {} {\mathscr {J}}^{\mathrm{RKKY}}_{\alpha \beta }(\mathbf{R })\, \mathbf{S }_1 \cdot \mathbf{S }_2\, , \end{aligned}$$

where ${\mathscr {J}}^{\mathrm{RKKY}}_{\alpha \beta }(\mathbf{R })$ is called the RKKY exchange interaction strength given by

12 $$\begin{aligned} {\mathscr {J}}^{\mathrm{RKKY}}_{\alpha \beta }(\mathbf{R }) = {\mathscr {J}}^2 \chi _{\alpha \beta }(0,\mathbf{R })\, , \end{aligned}$$

in which one of the impurities is set to the zero position and another one at $\mathbf{R }$. In this equation, $\chi _{\alpha \beta }(0,\mathbf{R })$ is the spin susceptibility which is directly proportional to the RKKY exchange interaction ${\mathscr {J}}^{\mathrm{RKKY}}_{\alpha \beta }(\mathbf{R })$.

The spin susceptibility for our spin-degenerate system described by a two-band model can be extracted using the retarded Green’s functions^48–51^ as

13 $$\begin{aligned} \chi ^{\alpha \beta }_{ij}(\,\mathbf{R }\,) = -\frac{2}{\pi } \mathrm {Im} \int ^{{\mathscr {E}}_{\mathrm {F}}}_{-\infty } d{\mathscr {E}}\, {\mathscr {C}}^{\alpha \beta }_{ij}({\mathscr {E}},\mathbf{R },0)\, , \end{aligned}$$

where $i,j \in \{x,y,z\}$, ${\mathscr {E}}_{\mathrm{F}}$ is the Fermi energy and

14 $$\begin{aligned} {\mathscr {C}}^{\alpha \beta }_{ij}({\mathscr {E}},\mathbf{R },0)=\mathrm {Tr}\, \left[ \sigma _{i} \, G^0_{\alpha \beta }({\mathscr {E}},\mathbf{R },0)\, \sigma _{j} \, G^0_{\beta \alpha }({\mathscr {E}},0,\mathbf{R }) \right] \, , \end{aligned}$$

wherein $G^0_{\alpha \beta }({\mathscr {E}},\mathbf{R },0)$ is the $2\times 2$ matrix of lattice non-interacting Green’s functions in the spin space. Regarding the Fermi energy, Fig. 3 states that it lies in the zero energy, in both gapless and gapped [middle of band gap] phases. To study the RKKY responses when the system is not doped by donors or acceptors, it is our task to set ${\mathscr {E}}_{\mathrm{F}}$ to zero in the following. Taking the Fourier transform of the reciprocal-space Green’s functions into account in the vicinity of two in-equivalent $X_1$ and $X_2$ points in the SBZ of (001) plane in TCIs, one obtains

15 $$\begin{aligned} G^0_{\alpha \beta }({\mathscr {E}},\mathbf{R },0) ={} \frac{1}{\Omega _{\mathrm {SBZ}}}\int d^2q\, e^{\texttt {i}\vec {q}\cdot \mathbf{R }} \Big [e^{\texttt {i}\vec {X}_1\cdot \mathbf{R }} G^0_{\alpha \beta }(\vec {q} + \vec {X}_1,{\mathscr {E}}) + e^{\texttt {i}\vec {X}_2\cdot \mathbf{R }} G^0_{\alpha \beta }(\vec {q} + \vec {X}_2,{\mathscr {E}})\Big ]\, , \end{aligned}$$

where $\vec {q}$ is the momenta around the Dirac cones. These momenta should be small enough so that each Dirac cone involves in the interaction, i.e. $|\vec {q}|\ll |\vec {\Lambda }_i|$ and $|\vec {\Lambda }'_i|$ ($i \in \{x,y\}$). Also, the integration is performed over the entire SBZ of $\Omega _{\mathrm {SBZ}}$ shown in Fig. 2a including four Dirac cones along different directions. Accordingly, one allows to rewrite the Eq. (15) as

16 $$\begin{aligned} G^0_{\alpha \beta }({\mathscr {E}},\mathbf{R },0) ={} e^{\mathtt{i}X_1\,R_x}\left( e^{\mathtt{i}\Lambda _x\,R_x}{\mathscr {G}}^{0,x}_{\alpha \beta }+e^{-\mathtt{i}\Lambda _x\,R_x}{\mathscr {G}}^{0,-x}_{\alpha \beta }\right) + e^{\mathtt{i}X_2\,R_y}\left( e^{\mathtt{i}\Lambda _y\,R_y}{\mathscr {G}}^{0,y}_{\alpha \beta }+e^{-\mathtt{i}\Lambda _y\,R_y}{\mathscr {G}}^{0,-y}_{\alpha \beta }\right) \, , \end{aligned}$$

with the defined $R_x = R\,\cos (\varphi _R)$ and $R_y = R\,\sin (\varphi _R)$. All ${\mathscr {G}}$ functions are the real-space Green’s function, however, the reciprocal-space Green’s function is easily calculated through

17 $$\begin{aligned} {\mathscr {G}}^{0,x}(q,{\mathscr {E}}) = {}\left[ {\mathscr {E}}+\mathtt{i}o^+ -{\mathscr {H}}_{\Lambda _x}(q,{\mathscr {E}})\right] ^{-1} ={} \frac{1}{\mathrm{det}} \begin{pmatrix} -\mathtt{i} \omega - \Delta _{x\mathrm{F}} &{} \mathtt{i} v_{\mathrm{F}} q e^{\mathtt{i}\varphi _q} \\ \\ -\mathtt{i} v_{\mathrm{F}} q e^{-\mathtt{i}\varphi _q} &{} -\mathtt{i} \omega + \Delta _{x\mathrm{F}} \end{pmatrix}={} \begin{pmatrix} {\mathscr {G}}^{0,x}_{11}(q,{\mathscr {E}}) &{} {\mathscr {G}}^{0,x}_{12}(q,{\mathscr {E}})\\ \\ {\mathscr {G}}^{0,x}_{21}(q,{\mathscr {E}}) &{} {\mathscr {G}}^{0,x}_{22}(q,{\mathscr {E}}) \end{pmatrix}\, , \end{aligned}$$

where ${\mathscr {E}}+\mathtt{i}o^+ = \mathtt{i} \omega$ and $\mathrm{det} = \omega ^2 + \Delta ^2_{x\mathrm{F}} + v^2_F q^2$. Using $d^2q = q\,dq\,d\varphi _q$ and $\exp \left[ \texttt {i}\vec {q}\cdot \mathbf{R }\right] =\exp \left[ \texttt {i}\,q\,R\,\cos \left( \varphi _q-\varphi _R\right) \right]$ with $\varphi _R=\tan ^{-1}\left( R_y/R_x\right)$ and $\varphi _q=\tan ^{-1}\left( q_y/q_x\right)$, one obtains

18 $$\begin{aligned} {\mathscr {G}}^{0,i}_{\alpha \beta }(\omega , \mathbf{R },0) ={} \frac{1}{\Omega _{\mathrm {SBZ}}} \int ^{q_c}_{0} q\,d q\,\int ^{2\pi }_{0} d\varphi _q {} e^{\texttt {i}\,q\,R\,\cos \left( \varphi _q-\varphi _R\right) }\, {\mathscr {G}}^{0,i}_{\alpha \beta }(q,\omega )\, . \end{aligned}$$

It is necessary to point out that we are allowed to extend the momentum cutoff $q_c$ to $\infty$ since the RKKY response at long (short) distance between magnetic moments arises mainly from the small (large) momenta. Thus, to cover the most contribution of momenta over the entire SBZ, we use $q_c \rightarrow \infty$ simply.

After pretty simple calculations, we find

19a $$\begin{aligned} {\mathscr {G}}^{0,x}_{11}(\omega , \mathbf{R },0) = {}&\frac{2\pi \left( -\mathtt{i} \omega -\Delta _{x\mathrm{F}}\right) }{\Omega _{\mathrm {SBZ}}\,v^2_{\mathrm{F}}} K_0\left( \frac{{\mathscr {A}}\,R}{v_F}\right) \, , \end{aligned}$$

19b $$\begin{aligned} {\mathscr {G}}^{0,x}_{12}(\omega , \mathbf{R },0) = {}&\frac{2\pi \,e^{\mathtt{i}\varphi _R}{\mathscr {A}}}{\Omega _{\mathrm {SBZ}}\,v^2_{\mathrm{F}}} K_1\left( \frac{{\mathscr {A}}\,R}{v_F}\right) \, , \end{aligned}$$

19c $$\begin{aligned} {\mathscr {G}}^{0,x}_{21}(\omega , \mathbf{R },0) = {}&\frac{2\pi \,e^{-\mathtt{i}\varphi _R}{\mathscr {A}}}{\Omega _{\mathrm {SBZ}}\,v^2_{\mathrm{F}}} K_1\left( \frac{{\mathscr {A}}\,R}{v_F}\right) \, , \end{aligned}$$

19d $$\begin{aligned} {\mathscr {G}}^{0,x}_{22}(\omega , \mathbf{R },0) = {}&\frac{2\pi \left( -\mathtt{i} \omega +\Delta _{x\mathrm{F}}\right) }{\Omega _{\mathrm {SBZ}}\,v^2_{\mathrm{F}}} K_0\left( \frac{{\mathscr {A}}\,R}{v_F}\right) \, , \end{aligned}$$

where ${\mathscr {A}} = \sqrt{\omega ^2 + \Delta ^2_{x\mathrm{F}}}$ and $K_{0/1}({\mathscr {A}}\,R/v_F)$ is the modified Bessel function of the zero/first kind. Note that these only provide the lattice Green’s functions for $\Lambda _x$ point. It is straightforward to deduce that

20a $$\begin{aligned} {\mathscr {G}}^{0,y}_{11}(\omega , \mathbf{R },0) = {}&\frac{2\pi \left( -\mathtt{i} \omega -\Delta _{y\mathrm{F}}\right) }{\Omega _{\mathrm {SBZ}}\,v^2_{\mathrm{F}}} K_0\left( \frac{{\mathscr {B}}\,R}{v_F}\right) \, , \end{aligned}$$

20b $$\begin{aligned} {\mathscr {G}}^{0,y}_{12}(\omega , \mathbf{R },0) = {}&\frac{2\pi \,e^{\mathtt{i}\varphi _R}{\mathscr {B}}}{\Omega _{\mathrm {SBZ}}\,v^2_{\mathrm{F}}} K_1\left( \frac{{\mathscr {B}}\,R}{v_F}\right) \, , \end{aligned}$$

20c $$\begin{aligned} {\mathscr {G}}^{0,y}_{21}(\omega , \mathbf{R },0) = {}&\frac{2\pi \,e^{-\mathtt{i}\varphi _R}{\mathscr {B}}}{\Omega _{\mathrm {SBZ}}\,v^2_{\mathrm{F}}} K_1\left( \frac{{\mathscr {B}}\,R}{v_F}\right) \, , \end{aligned}$$

20d $$\begin{aligned} {\mathscr {G}}^{0,y}_{22}(\omega , \mathbf{R },0) = {}&\frac{2\pi \left( -\mathtt{i} \omega +\Delta _{y\mathrm{F}}\right) }{\Omega _{\mathrm {SBZ}}\,v^2_{\mathrm{F}}} K_0\left( \frac{{\mathscr {B}}\,R}{v_F}\right) \, , \end{aligned}$$

where $${\mathscr {B}} = \sqrt{\omega ^2 + \Delta ^2_{y\mathrm{F}}}$$.

By inspiration of the two-fold ${\mathscr {C}}_2$ and four-fold ${\mathscr {C}}_4$ rotational symmetries, one simply finds lattice Green’s functions for other three Dirac points:

21a $$\begin{aligned} {\mathscr {G}}^{0,-x}(\omega , \mathbf{R },0) = {}&\begin{pmatrix} {\mathscr {G}}^{0,x}_{22}(\omega , \mathbf{R },0) &{} {\mathscr {G}}^{0,x}_{12}(\omega , \mathbf{R },0)\\ \\ {\mathscr {G}}^{0,x}_{21}(\omega , \mathbf{R },0) &{} {\mathscr {G}}^{0,x}_{11}(\omega , \mathbf{R },0) \end{pmatrix}\, , \end{aligned}$$

21b $$\begin{aligned} {\mathscr {G}}^{0,y}(\omega , \mathbf{R },0) = {}&\begin{pmatrix} {\mathscr {G}}^{0,y}_{11}(\omega , \mathbf{R },0) &{} {\mathscr {G}}^{0,y}_{12}(\omega , \mathbf{R },0)\\ \\ {\mathscr {G}}^{0,y}_{21}(\omega , \mathbf{R },0) &{} {\mathscr {G}}^{0,y}_{22}(\omega , \mathbf{R },0) \end{pmatrix}\, , \end{aligned}$$

21c $$\begin{aligned} {\mathscr {G}}^{0,-y}(\omega , \mathbf{R },0) = {}&\begin{pmatrix} {\mathscr {G}}^{0,y}_{22}(\omega , \mathbf{R },0) &{} {\mathscr {G}}^{0,y}_{12}(\omega , \mathbf{R },0)\\ \\ {\mathscr {G}}^{0,y}_{21}(\omega , \mathbf{R },0) &{} {\mathscr {G}}^{0,y}_{11}(\omega , \mathbf{R },0) \end{pmatrix}\, . \end{aligned}$$

Substituting Eqs. (21) and (19) into the Eq. (16), the retarded Green’s functions read as

22a $$\begin{aligned}&G^0_{11}(\omega ,\mathbf{R },0) ={} e^{\mathtt{i}X_1\,R_x}\left( e^{\mathtt{i}\Lambda _x\,R_x}{\mathscr {G}}^{0,x}_{11}+e^{-\mathtt{i}\Lambda _x\,R_x}{\mathscr {G}}^{0,x}_{22}\right) +{} e^{\mathtt{i}X_2\,R_y}\left( e^{\mathtt{i}\Lambda _y\,R_y}{\mathscr {G}}^{0,y}_{11}+e^{-\mathtt{i}\Lambda _y\,R_y}{\mathscr {G}}^{0,y}_{22}\right) \, , \end{aligned}$$

22b $$\begin{aligned}&G^0_{12}(\omega ,\mathbf{R },0) ={} e^{\mathtt{i}X_1\,R_x}\left( e^{\mathtt{i}\Lambda _x\,R_x}{\mathscr {G}}^{0,x}_{12}+e^{-\mathtt{i}\Lambda _x\,R_x}{\mathscr {G}}^{0,x}_{12}\right) +{}e^{\mathtt{i}X_2\,R_y}\left( e^{\mathtt{i}\Lambda _y\,R_y}{\mathscr {G}}^{0,y}_{12}+e^{-\mathtt{i}\Lambda _y\,R_y}{\mathscr {G}}^{0,y}_{12}\right) \, , \end{aligned}$$

22c $$\begin{aligned}&G^0_{21}(\omega ,\mathbf{R },0) ={} e^{-\mathtt{i}X_1\,R_x}\left( e^{-\mathtt{i}\Lambda _x\,R_x}{\mathscr {G}}^{0,x}_{21}+e^{\mathtt{i}\Lambda _x\,R_x}{\mathscr {G}}^{0,x}_{21}\right) +{} e^{-\mathtt{i}X_2\,R_y}\left( e^{-\mathtt{i}\Lambda _y\,R_y}{\mathscr {G}}^{0,y}_{21}+e^{\mathtt{i}\Lambda _y\,R_y}{\mathscr {G}}^{0,y}_{21}\right) \, , \end{aligned}$$

22d $$\begin{aligned}&G^0_{22}(\omega ,\mathbf{R },0) ={} e^{\mathtt{i}X_1\,R_x}\left( e^{\mathtt{i}\Lambda _x\,R_x}{\mathscr {G}}^{0,x}_{22}+e^{-\mathtt{i}\Lambda _x\,R_x}{\mathscr {G}}^{0,x}_{11}\right) +{} e^{\mathtt{i}X_2\,R_y}\left( e^{\mathtt{i}\Lambda _y\,R_y}{\mathscr {G}}^{0,y}_{22}+e^{-\mathtt{i}\Lambda _y\,R_y}{\mathscr {G}}^{0,y}_{11}\right) \, . \end{aligned}$$

Turning to the Eq. (14) gives rise to ${\mathscr {C}}^{\alpha \beta }_{ii}(\omega ,\mathbf{R },0) = G^0_{\alpha \beta }(\omega ,\mathbf{R },0) G^0_{\alpha \beta }(\omega ,0,\mathbf{R })$ and ${\mathscr {C}}^{\alpha \beta }_{ij}(\omega ,\mathbf{R },0) = 0$ which *i* and *j* could be each of *x*, *y* and *z* due to the spin-degeneracy. Eventually, using the feature $d{\mathscr {E}} = \mathtt{i} d \omega$ from the definition of ${\mathscr {E}}+\mathtt{i}o^+ = \mathtt{i} \omega$, the spin susceptibility reads

23 $$\begin{aligned} \chi ^{\alpha \beta }_{ii}(\,\mathbf{R }\,) = -\frac{2}{\pi } \int ^{0}_{-\infty } d\omega \,\mathfrak {R}\left[ G^0_{\alpha \beta }(\omega ,\mathbf{R },0)\,G^0_{\alpha \beta }(\omega ,0,\mathbf{R })\right] \, , \end{aligned}$$

Having Green’s functions as well as susceptibility we divide the following discussion into two parts: (i) impurities located on the same sublattices and (ii) impurities located on different sublattices. We comment that it is of course possible to locate impurities on the center of square plaquettes or on the bonds of the SnTe (001) surface. For impurities far-enough from each other n compared to lattice parameter, the Hamiltonian can be simply given by ${\mathscr {H}}_{\mathrm{int}} = - {\mathscr {J}} (\vec {S}_1 \cdot \sum _{\mathrm{e}} s_{\mathrm{e}} + \vec {S}_2 \cdot \sum _{\mathrm{e'}} s_{\mathrm{e'}})$ where *e* and $e'$ are the itinerant electrons living around nearest neighbor magnetic impurities $\vec {S}_1$ and $\vec {S}_2$. By this, we respectively find $\chi ^{\mathrm{center}}_{ii}(\,\mathbf{R },0\,) = 8\,[\chi ^{\alpha \alpha }_{ii}(\,\mathbf{R },0\,) + \chi ^{\alpha \beta }_{ii}(\,\mathbf{R },0\,)]$ and $\chi ^{\mathrm{bond}}_{ii}(\,\mathbf{R },0\,) = 2\,[\chi ^{\alpha \alpha }_{ii}(\,\mathbf{R },0\,) + \chi ^{\alpha \beta }_{ii}(\,\mathbf{R },0\,)]$ when the impurities are located at the center of square plaquettes and bonds between nearest neighbors. It is clear that having the responses on the same and different sublattices, these two extra cases can easily be extracted. The stronger response of impurities on different sublattices [it will be confirmed later] is dominant to calculate the entire response of these two extra configurations. So the magnetic susceptibility will show an antiferromagnetic ordering in the system. However, we would neglect these configurations in the present paper from a pragmatic point of view. Also, the bulk states do not significantly affect the RKKY coupling because it has been found that although distortion has a negligible effect on the band structure in the bulk, it can dramatically affect the Dirac surface states^7^. We note that Dirac materials that made a revolution in condensed matter physics have a strong impact on magnetic couplings. The decaying rate of RKKY coupling is faster in Dirac materials compared to 2D ordinary metals^24–28,30–34^. This faster decaying is also seen for gapped Dirac cones. In other words, gapped Dirac cones have different results than other gapped semiconductors.

### Impurities on the same sublattices

When two magnetic moments reside on the same sublattices of the surface (001) of SnTe (the same Sn or Te sublattices), the susceptibility can be calculated as

24 $$\begin{aligned} \chi ^{\alpha \alpha }_{ii}(\,\mathbf{R }\,) = -\frac{2}{\pi } \int ^{0}_{-\infty } d\omega \,\mathfrak {R}\left[ G^0_{\alpha \alpha }(\omega ,\mathbf{R },0)\,G^0_{\alpha \alpha }(\omega ,0,\mathbf{R })\right] \, , \end{aligned}$$

After tedious calculations and defining $\gamma = X_1 R_x - X_2 R_y$, $\xi = \Lambda _x R_x - \Lambda _y R_y$ and $\zeta = \Lambda _x R_x + \Lambda _y R_y$, we achieve

25 $$\begin{aligned} \begin{aligned} \frac{\chi ^{\alpha \alpha }_{ii}(\,\mathbf{R }\,,\varphi _R)}{C_t} = {}&\int ^{0}_{-\infty } d\omega \, (\omega ^2-\Delta ^2_{x\mathrm{F}})K^2_0\left( {\mathscr {A}}R/v_{\mathrm{F}}\right) \\&+C_1 \int ^{0}_{-\infty } d\omega \, (\omega ^2+\Delta ^2_{x\mathrm{F}})K^2_0\left( {\mathscr {A}}R/v_{\mathrm{F}}\right) + \int ^{0}_{-\infty } d\omega \, (\omega ^2-\Delta ^2_{y\mathrm{F}})K^2_0\left( {\mathscr {B}}R/v_{\mathrm{F}}\right) \\ {}&+C_2 \int ^{0}_{-\infty } d\omega \, (\omega ^2+\Delta ^2_{y\mathrm{F}})K^2_0\left( {\mathscr {B}}R/v_{\mathrm{F}}\right) +C_3 \int ^{0}_{-\infty } d\omega \, (\omega ^2-\Delta _{x\mathrm{F}}\,\Delta _{y\mathrm{F}})K_0\left( {\mathscr {A}}R/v_{\mathrm{F}}\right) \,K_0\left( {\mathscr {B}}R/v_{\mathrm{F}}\right) \\ {}&+C_4 \int ^{0}_{-\infty } d\omega \, (\omega ^2+\Delta _{x\mathrm{F}}\,\Delta _{y\mathrm{F}})K_0\left( {\mathscr {A}}R/v_{\mathrm{F}}\right) \,K_0\left( {\mathscr {B}}R/v_{\mathrm{F}}\right) \, , \end{aligned} \end{aligned}$$

where $C_t = 16\pi /\Omega ^2_{\mathrm {SBZ}}v^4_{\mathrm{F}}$, $C_1 = \cos (2\Lambda _x\,R_x)$, $C_2 = \cos (2\Lambda _y\,R_y)$, $C_3 = 2\cos \gamma \cos \xi$, and $C_4 = 2\cos \gamma \cos \zeta$. We comment that the susceptibility is function of both distance *R* and the angel $\varphi _R$ due to the presence of $\{R_x,R_y\}$. Also, we stress that the RKKY interaction between two magnetic moments on the same Sn and/or Te sublattices is the same, i.e. $\chi ^{11}_{ii}(\,\mathbf{R }\,,\varphi _R) = \chi ^{22}_{ii}(\,\mathbf{R }\,,\varphi _R)$. Simply, it is straightforward to conclude that the coefficients $C_i$ [$i\in {1,2,3,4}$] are the origins of the sign changing in the susceptibility in the presence of the distortion.

**Figure 4:**
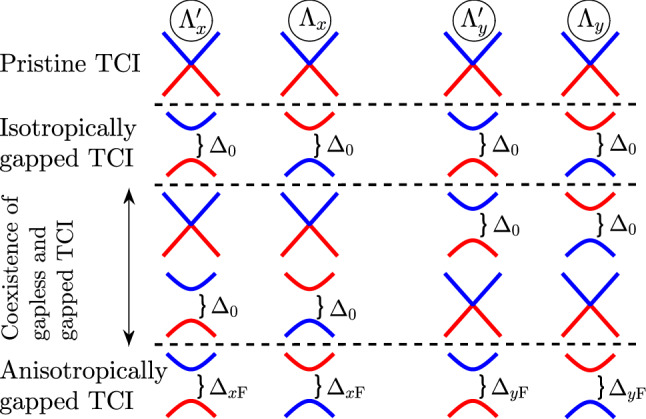
Two-band energy spectrum of four possible configurations for the gapes $\Delta _{x \mathrm F}$ and $\Delta _{y \mathrm F}$, namely pristine TCI, isotropically gapped TCI, coexistence of gapless and gapped TCI, and anisotropically gapped TCI.

As shown in Eq. (25), the spin susceptibility when the magnetic moments reside on the same sublattices supports a wide range of possibilities on the spin flipping and eventually on the magnetic phase transitions, which strongly depend on the gap strengths induced by $\Delta _{x\mathrm{F}}$ and $\Delta _{y\mathrm{F}}$. To reveal the ferroelectric distortion effect on the RKKY interaction ${\mathscr {J}}^{\mathrm{RKKY}}_{\alpha \alpha }(\mathbf{R }, \varphi _R)/ {\mathscr {J}}^2\,C_t = \chi ^{\alpha \alpha }_{ii}(\,\mathbf{R }\,,\varphi _R)/C_t$, we consider different possible configurations. Thus, to deduce the clear physical insights from such a complex response, we divide the following analysis into four parts, as represented in Fig. 4 namely

1. Pristine TCI
2. Isotropically gapped TCI
3. Coexistence of gapless and gapped TCI
4. Anisotropically gapped TCI

Particularly, this classification is necessary due to various possible configurations for the gaps $\Delta _{x\mathrm{F}}$ and $\Delta _{y\mathrm{F}}$. The specific nature of each part will be focused on the *magnetic ordering* and *the role of short- and long-range* responses. By this explicit elucidation, one can easily distinguish the novelty of electronic and/or magnetic features in TCIs compared to other gapless and gapped Dirac materials.

For the magnetic ordering, the sign of susceptibility helps, however, in all cases, it is not hard to seek for the short- and long-range behaviors of the RKKY response. It can be simply formulated considering the conditions $\Delta _{x/y\mathrm{F}} R/v_{\mathrm{F}} \ll 1$ and $\Delta _{x/y\mathrm{F}} R/v_{\mathrm{F}} \gg 1$ in Eq. (25). By this, using the simplified modified Bessel functions

26a $$\begin{aligned} \lim _{z\rightarrow 0} K_{\nu }(z) \sim {}&{\left\{ \begin{array}{ll} -\ln (\frac{z}{2}) - \gamma ' &{} \hbox { if}\ \nu = 0\\ \frac{1}{2}\,\Gamma (\nu )\,(\frac{z}{2})^{-\nu } &{} \hbox { if}\ \nu >0 \end{array}\right. }\, , \end{aligned}$$

26b $$\begin{aligned} \lim _{z\rightarrow \infty } K_{\nu }(z) \sim {}&\sqrt{\frac{\pi }{2\,z}}\,e^{-z}\, , \end{aligned}$$

with $\gamma ' = 0.5772$ being the Euler-Mascheroni constant, one finds corresponding short- and long-range responses.

**Figure 5:**
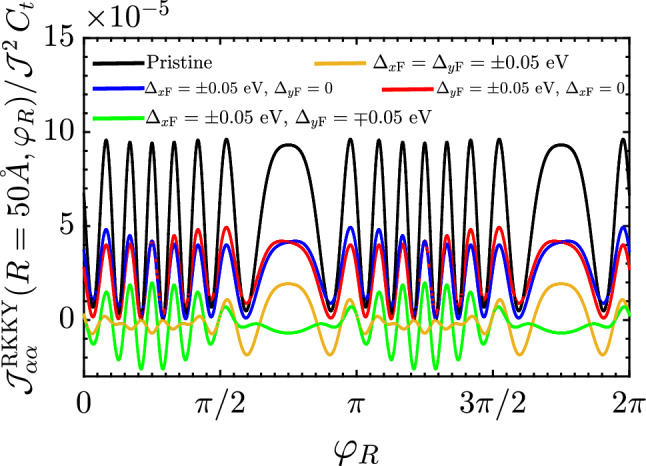
The behavior of the RKKY interaction ${\mathscr {J}}^{\mathrm{RKKY}}_{\alpha \alpha }(\mathbf{R }, \varphi _R)/ {\mathscr {J}}^2\,C_t$ as a function of $\varphi _R$ at fixed $R = 50$ Å  for different ferroelectric distortion strengths. The obvious periodic feature of the RKKY coupling in the pristine and distorted square lattice of the SnTe (001) surface is given by ${\mathscr {J}}^{\mathrm{RKKY}}_{\alpha \alpha }(\mathbf{R }, \varphi _R) = {\mathscr {J}}^{\mathrm{RKKY}}_{\alpha \alpha }(\mathbf{R }, \varphi _R + 2\pi )$.

Before turning to the analysis of the results, we would focus on the periodic feature of the RKKY coupling in the pristine and distorted square lattice of the SnTe (001) surface. By the lattice, one easily requires the property ${\mathscr {J}}^{\mathrm{RKKY}}_{\alpha \alpha }(\mathbf{R }, \varphi _R) = {\mathscr {J}}^{\mathrm{RKKY}}_{\alpha \alpha }(\mathbf{R }, \varphi _R + 2\pi )$ at any distance *R* and any gap, as confirmed by the numerical result represented in Fig. 5, however, different interaction strengths between two magnetic impurities at different positionings are obvious. This anisotropy of the susceptibility could be expected from the cosinusoidal functions (included in $C_i$ coefficients) behind the integrals in Eq. (25). A general feature in the RKKY response is the gap-dependent beating pattern with distinct periodicities, meaning that various Fermi wavelengths contribute to form the entire periodic pattern. In our system, multiple surface Dirac cones, two along the *x* direction and two along the *y* direction in the SBZ of SnTe (001) and related alloys are responsible for the beating effect in the RKKY oscillations. Now it is time to study each aforementioned part.

#### Pristine TCI

For the pristine case, i.e. $\Delta _{x\mathrm{F}} = \Delta _{y\mathrm{F}} = 0$ [see the first row of Fig. 4], following Eq. (25) we achieve the expression

27 $$\begin{aligned} \frac{\chi ^{\alpha \alpha }_{ii}(\,\mathbf{R }\,,\varphi _R)}{C_t} = {} 2\,{\mathscr {F}}(\mathbf{R }\,,\varphi _R)\,\underbrace{\int ^{0}_{-\infty } d\omega \, \omega ^2\,K^2_0\left( \omega R/v_{\mathrm{F}}\right) }_{\pi ^2 v^3_{\mathrm{F}}/32 R^3} {}\propto \frac{{\mathscr {F}}(\mathbf{R }\,,\varphi _R)}{R^3}\, , \end{aligned}$$

where

28 $$\begin{aligned} {\mathscr {F}}(\mathbf{R }\,,\varphi _R)={} 2 + C_1 + C_2 + C_3 + C_4 ={} \cos ^2(\Lambda _x\,R_x)+\cos ^2(\Lambda _y\,R_y){}+2\,\cos \gamma \,\cos (\Lambda _x\,R_x)\,\cos (\Lambda _y\,R_y)\, , \end{aligned}$$

in great agreement with our previous work^43^. This clearly shows that the term ${\mathscr {F}}(\mathbf{R }\,,\varphi _R)$ behind the integrals causes the positive sign of the susceptibility forever, meaning that there is no magnetic phase transition at all and the *pristine* system possesses a ferromagnetic ordering.

**Figure 6:**
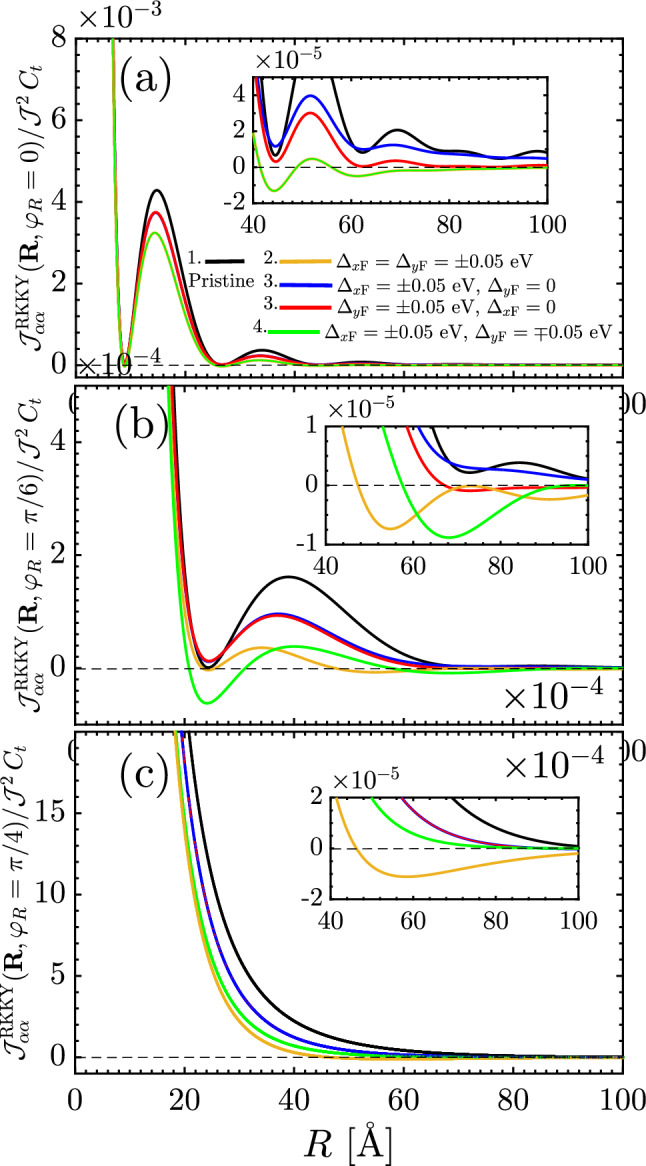
The behavior of the RKKY interaction ${\mathscr {J}}^{\mathrm{RKKY}}_{\alpha \alpha }(\mathbf{R }, \varphi _R)/ {\mathscr {J}}^2\,C_t$ as a function of *R* for different ferroelectric distortion potentials at (**a**) $\varphi _R=0$, (**b**) $\varphi _R=\pi /6$ and (**c**) $\varphi _R=\pi /4$.

Moreover, the RKKY response oscillates with *R* because of the cosine functions in the interference term ${\mathscr {F}}(\mathbf{R }\,,\varphi _R)$. On the other hand, since the integral is solvable analytically and no limitation is needed, one observes the decaying rate of the susceptibility serving as $R^{-3}$ in both short- and long-range impurity separations. The decaying rate treatment is similar to that of undoped graphene^48–51^. All these are understandable in all black curves in Fig. 6a–c, for which the response shows a ferromagnetic spin arrangement due to the positive sign of RKKY interaction. We avoid the repetition of the results of this part since they are well-established in Ref.^43^.

#### Isotropically gapped TCI

To determine the susceptibility of isotropically gap-induced TCIs due to the isotropic ferroelectric distortion, we consider $|\Delta _{x\mathrm{F}}| = |\Delta _{y\mathrm{F}}| = \Delta _0$ [see the second row of Fig. 4]. We would stress that for the case of proximity coupling to a ferromagnet, a mass term with the same signs at $\Lambda _x$ and $\Lambda '_x$, is induced by the exchange field on the SnTe (001) surface to align the spin direction. Or it may be regarded as the Zeeman term arising from a perpendicular external magnetic field^40^. However, the theory of the gapped Dirac model presented in this work, refers to a sign change of mass terms for the closed Dirac cones. In this case, combining the coefficients behind the integrals in Eq. (25) leads to the expression

29 $$\begin{aligned} \frac{\chi ^{\alpha \alpha }_{ii}(\,\mathbf{R }\,,\varphi _R)}{C_t} = {} 2\,{\mathscr {F}}(\mathbf{R }\,,\varphi _R)\,\int ^{0}_{-\infty } d\omega \, \omega ^2\,K^2_0\left( {\mathscr {A}}' R/v_{\mathrm{F}}\right) + {} 2\,{\mathscr {G}}(\mathbf{R }\,,\varphi _R)\,\Delta ^2_0\,\int ^{0}_{-\infty } d\omega \,K^2_0\left( {\mathscr {A}}' R/v_{\mathrm{F}}\right) \, , \end{aligned}$$

where $${\mathscr {A}}' = \sqrt{\omega ^2 + \Delta ^2_0}$$ and

30 $$\begin{aligned} {\mathscr {G}}(\mathbf{R }\,,\varphi _R)={} C_1 + C_2 + C_4 -2 - C_3 ={} \cos ^2(\Lambda _x\,R_x)+\cos ^2(\Lambda _y\,R_y){}-2\,\cos \gamma \,\sin (\Lambda _x\,R_x)\,\sin (\Lambda _y\,R_y) - 2\, . \end{aligned}$$

Regarding the magnetic ordering of this configuration, we comment that both ferromagnetic and antiferromagnetic phases are possible to emerge because of the mixture of sine and cosine functions in the interference terms behind the integrals. However, to elucidate the role of short- and long-range impurity separations, we stick to Eq. (26) as well as we use the mathematical identity^52^

31 $$\begin{aligned} \int _{-\infty }^0 x^{\alpha -1}K_{\mu }(c\,x)\,K_{\nu }(c\,x) dx = {} \frac{2^{\alpha -3}}{c^\alpha \Gamma (\alpha )} \Gamma \left( \frac{\alpha +\mu +\nu }{2}\right) {}\Gamma \left( \frac{\alpha +\mu -\nu }{2}\right) \Gamma \left( \frac{\alpha -\mu +\nu }{2}\right) \Gamma \left( \frac{\alpha -\mu -\nu }{2}\right) \,, \end{aligned}$$

resulting in the final expressions respectively for the short-range $\Delta _0R/v_{\mathrm{F}}\ll 1$

32 $$\begin{aligned} \begin{aligned} 
\frac{\chi ^{\alpha \alpha }_{ii}(\,\mathbf{R }\,,\varphi _R)}{C_t} \propto {} \frac{{\mathscr {F}}(\mathbf{R }\,,\varphi _R)}{R^3}\, , \end{aligned} \end{aligned}$$

and for the long-range $\Delta _0R/v_{\mathrm{F}}\gg 1$ with the help of Laplace method

33 $$\begin{aligned} \begin{aligned} \frac{\chi ^{\alpha \alpha }_{ii}(\,\mathbf{R }\,,\varphi _R)}{C_t} \propto {} \frac{{\mathscr {G}}(\mathbf{R }\,,\varphi _R)\,\Delta ^{3/2}_0}{R^{3/2}}\,e^{-2\,\Delta _0\,R/v_{\mathrm{F}}}\, . \end{aligned} \end{aligned}$$

Thus the short-range responses demonstrate the ferromagnetic ordering (stemming from the interference term ${\mathscr {F}}(\mathbf{R }\,,\varphi _R)$ in which the direction $\varphi _R$ is not important in changing the sign of the susceptibility) with the decaying rate of $R^{-3}$ like the pristine TCIs, while the long-range response decays as $R^{-3/2}\Delta ^{3/2}_0\,e^{-2\,\Delta _0\,R/v_{\mathrm{F}}}$ and both ferromagnetic and antiferromagnetic orderings are expected to appear due to the interference term ${\mathscr {G}}(\mathbf{R }\,,\varphi _R)$ in which the direction $\varphi _R$ is a matter in sign switching of the susceptibility. It is worthwhile mentioning that the RKKY interaction can not be influenced at all with the gap at short-range distances. The latter, in turn, means that the decaying function is a function associated with an extremum at which the critical distance *R* or gap $\Delta _0$ can be characterized. We will come to this last point later. The phase of the RKKY oscillations in metals becomes random in the presence of static non-magnetic impurities. In this case, averaging over various impurity configurations leads to an exponential factor appearing at distances larger than the mean free path^53,54^. An exponential decay on averaged RKKY couplings was also reported in disordered graphene^55^. In gapped graphene, RKKY coupling also experiences an exponential decay at large distances giving rise to Heisenberg interaction^48,56,57^.

**Figure 7:**
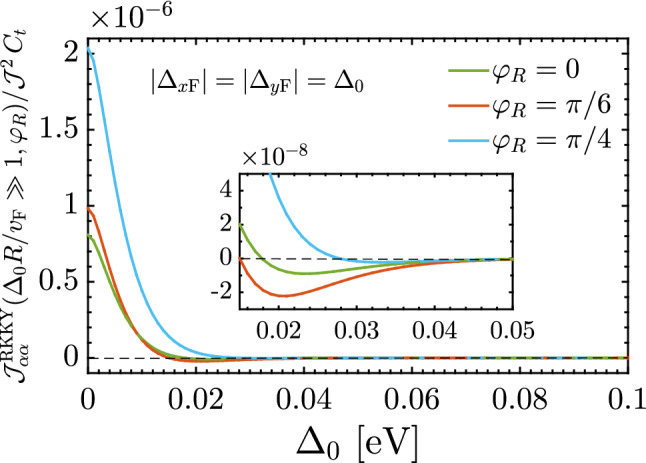
Long-range behavior of the RKKY interaction ${\mathscr {J}}^{\mathrm{RKKY}}_{\alpha \alpha }(\Delta _0R/v_{\mathrm{F}}\gg 1, \varphi _R)/ {\mathscr {J}}^2\,C_t$ as a function of $\Delta _0$ at fixed long-range impurity separation $R = 200$ Å  for different directions $\varphi _R$. It is evident that the RKKY response is an exponentially decaying function independent of $\varphi _R$ and there is a minimal gap at which the response starts to switch its trend.

In this configuration which the distortions are applied with the same strengths along different axes, referring as orange curves in our numerical results shown in Fig. 6a–c, the short-range treatment is independent of the gap, as expected from Eq. (32), while long-range one is associated with interesting spatial-dependent magnetic phase transitions expected from Eq. (33). Note that, physically point of view, the atomic packing factor for the same sublattices on the SnTe (001) surface along the *x* or *y* direction, Fig. 6a, is smaller than the atomic packing factor along the different directions, Fig. 6b and c. Thus, the shorter accessible distances are expected for $\varphi _R=0$. Although the short-range response is gap-independent, to describe long-range gap-dependent behaviors, we numerically plot Fig. 7 based on Eq. (25) at large-enough $R = 200$ Å  as a function of the gap potential $\Delta _0$. Interestingly, the feature concluded in Eq. (33) comes up, implying that the RKKY response is an exponentially decaying function along all directions $\varphi _R$ with a minimal gap at which the response starts to switch its trend [see the inset panel of Fig. 7].

#### Coexistence of gapless and gapped TCI

For the individual case of $|\Delta _{x\mathrm{F}}| = \Delta _0$ and $|\Delta _{y\mathrm{F}}| = 0$ [see the third and fourth rows of Fig. 4] we have

34 $$\begin{aligned} \begin{aligned} \frac{\chi ^{\alpha \alpha }_{ii}(\,\mathbf{R }\,,\varphi _R)}{C_t} = {}&(1+C_1)\,\int ^{0}_{-\infty } d\omega \, \omega ^2\,K^2_0\left( {\mathscr {A}}' R/v_{\mathrm{F}}\right) + (1+C_2)\,\int ^{0}_{-\infty } d\omega \, \omega ^2\,K^2_0\left( \omega R/v_{\mathrm{F}}\right) \\&+ {} (C_1-1)\,\Delta ^2_0\,\int ^{0}_{-\infty } d\omega \,K^2_0\left( {\mathscr {A}}' R/v_{\mathrm{F}}\right) \\{}&+ (C_3+C_4)\,\int ^{0}_{-\infty } d\omega \,\omega ^2\,K_0\left( \omega R/v_{\mathrm{F}}\right) \,K_0\left( {\mathscr {A}}' R/v_{\mathrm{F}}\right) \, . \end{aligned} \end{aligned}$$

The same expression can be obtained for the case of $|\Delta _{y\mathrm{F}}| = \Delta _0$ and $|\Delta _{x\mathrm{F}}| = 0$ subsituating $C_2 \mapsto C_1$ and $C_1 \mapsto C_2$ in the third first terms. Following Eqs. (26) and (31), we obtain respectively the effective short-range $\Delta _0R/v_{\mathrm{F}}\ll 1$ and long-range $\Delta _0R/v_{\mathrm{F}}\gg 1$ responses

35a $$\begin{aligned} \frac{\chi ^{\alpha \alpha }_{ii}(\,\mathbf{R }\,,\varphi _R)}{C_t} \propto {}&\frac{{\mathscr {F}}(\mathbf{R }\,,\varphi _R)}{R^3}\, , \end{aligned}$$

35b $$\begin{aligned} \frac{\chi ^{\alpha \alpha }_{ii}(\,\mathbf{R }\,,\varphi _R)}{C_t} \propto {}&\frac{(C_1-1)\,\Delta ^{3/2}_0}{R^{3/2}}\,e^{-2\,\Delta _0\,R/v_{\mathrm{F}}}\, , \end{aligned}$$

wherein the decaying rates are similar to the previous case, whereas the interference term for the long-range case is quite different. While depending on the $C_i$ coefficients, one expects almost the same behaviors for the RKKY interaction of gapped TCI along the *x* or *y* direction at short-range distances, the $\varphi _R$-dependent long-range interaction would be different [see blue and red curves in Fig. 6a–c].

**Figure 8:**
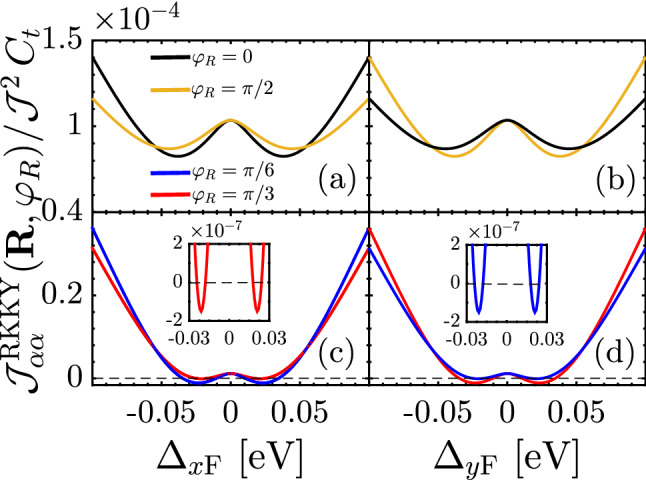
The behavior of the RKKY interaction ${\mathscr {J}}^{\mathrm{RKKY}}_{\alpha \alpha }(\mathbf{R }, \varphi _R)/ {\mathscr {J}}^2\,C_t$ as a function of ferroelectric distortion potential (**a**), (**c**) $\Delta _{x\mathrm{F}}$ and (**b**), (**d**) $\Delta _{y\mathrm{F}}$ for four different complementary angels $\varphi _R$ between two magnetic moments at $R = 25$ Å. The nontrivial behavior of RKKY response characterized by the decreasing and increasing trends is present as long as the gapless and gapped states are coexisted.

This part provides the first interesting novel remark of the current paper for which the SnTe (001) surface confronts two phases at the same time, a gapless Dirac cones along the *x*/*y* direction and a gapped one along the *y*/*x* direction. As mentioned in the introduction, multiple surface Dirac conses in different topological materials may show such an interesting feature as well^12–15^. However, the effects of such coexisted gapless and gapped phases on RKKY response can be rarely found in other Dirac materials. Thereby, one expects nontrivial behavior of the RKKY response.

In Eq.  (35), we found the underlying analytical expressions behind the short- and long-range responses. For the sake of completeness, it is necessary to discuss the intermediate-range RKKY response as well. Following the above points and considering structural symmetry of the SnTe (001) surface, one can expect some spatial symmetries between the results of $\Delta _{x\mathrm{F}}$ and $\Delta _{y\mathrm{F}}$ when they are present *individually*, resulting in the coexistence of gapless and gapped phases in TCIs. Also, the same expectation is valid for the complementary angles between magnetic impurities. To this end, we print Fig. 8 in which the RKKY response to the direction-dependent distortion potentials is plotted for two set of complementary angles, namely $\varphi _R = \{0,\pi /2\}$ and $\varphi _R =\{\pi /6,\pi /3\}$. It is necessary to note that in the pristine case, the mentioned complementary angles show the same susceptibilities, while the distortion breaks the symmetry between them down. Depending on the values of gaps, they may compete. Interestingly, it is evident that regarding the ${\mathscr {C}}_4$ rotation symmetry valid for pristine TCIs, the RKKY response to the $\Delta _{x\mathrm{F}}$ for $\varphi _R$ behaves similar to the case if it is studied in terms of $\Delta _{y\mathrm{F}}$ for $\pi /2-\varphi _R$ [see respectively panels {(a),(b)} and {(c),(d)}]. Although the magnetic impurities along the *x*/*y* direction illustrate no transition, the other angles show the phase transition at certain gaps [see inset panels in (c) and (d)]. Another remarkable point here refers to the gap sign effect on the RKKY response. The sign of $\Delta _{x\mathrm{F}}$ and $\Delta _{y\mathrm{F}}$ is not matter here and the RKKY response is symmetric concerning opposite gap signs with the fact that the susceptibility can not be influenced with the band inversion caused by the gap signs [please see once more the third and fourth rows of Fig. 4]. From this point, one would expect the same responses for ${\mathscr {J}}^{\mathrm{RKKY}}_{\alpha \alpha }(\mathbf{R }, \varphi _R)/ {\mathscr {J}}^2\,C_t$ with $\pm \Delta _{x\mathrm{F}}$ and $\pm \Delta _{y\mathrm{F}}$.

In addition to the above-mentioned points, we intend to discuss the origin of the decreasing and increasing trend of the susceptibility with the gap, which is that of nontrivial point reported before. In Fig. 8, the susceptibility decreases with $|\Delta _{x/y\mathrm{F}}|$ and after a critical gap potential, which is obviously *R*- and $\varphi _R$-dependent, increases. The reason can be traced back to the metallic phase of the system in one direction, while the gapped phase along another one. This implies that the reason for the observed nontrivial trend can be understood from the fact that the states around the band edges for the gapped Dirac cones also matter. These states which belong to the valence band, contribute to the RKKY interaction. The susceptibility is intensified if one can enhance the density of the band edge states in the valence band.

**Figure 9:**
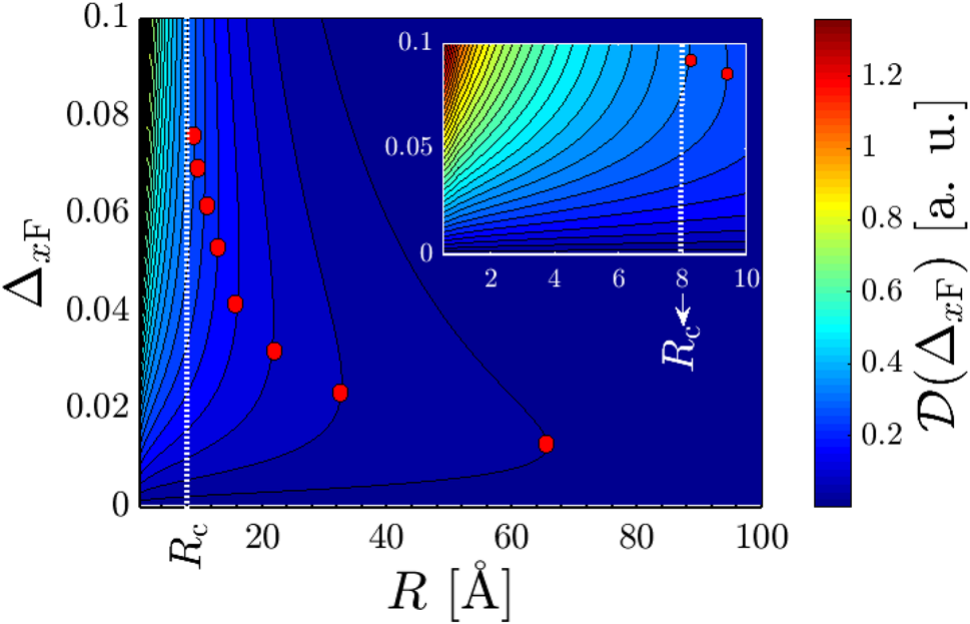
The behavior of the correlation function at ${\mathscr {E}} = \Delta _{x\mathrm{F}}$ as a function of ferroelectric distortion potential $\Delta _{x\mathrm{F}}$ and distance *R* for $\varphi _R = 0$ at $\Delta _{y\mathrm{F}}=0$. The critical gap for which ${\mathscr {D}}(\Delta _{x\mathrm{F}})$ turns to the increasing trend at a certain $R > R_{\mathrm{c}}$ decreases with the distance *R* labeled by red solid dots.

Indeed, the susceptibility is proportional to the correlation function defining as ${\mathscr {D}}_0 (\omega , \mathbf{R }) = -\tfrac{1}{\pi } \mathrm{Im} {\mathscr {G}}^0_{\alpha \alpha }(\omega , \mathbf{R },0)$. To this end, we stick ${\mathscr {D}}_0 (\omega , \mathbf{R })$ at the energies equal to the gap size, e.g. $\Delta _{x\mathrm{F}}$ [the analysis is precisely the same for $\Delta _{y\mathrm{F}}$] in Fig. 9 setting $\varphi _R = 0$ [as the representative test case] and $\Delta _{y\mathrm{F}}=0$. We intend to find the relation between the correlation function ${\mathscr {D}}(\Delta _{x\mathrm{F}}) = {\mathscr {D}}_0 (\omega = \Delta _{x\mathrm{F}})$ and $\{\Delta _{x\mathrm{F}},R\}$ to understand the physical reason behind the nontrivial behavior of the susceptibility in Fig. 8. Two qualitatively different regions as a function of the distance *R* may be seen: For short-range $R < R_{\mathrm{c}}$ [$R_{\mathrm{c}} = 8$ Å] a high ${\mathscr {D}}(\Delta _{x\mathrm{F}})$ is observed, which on increasing the distance decreases gradually for all $R > R_{\mathrm{c}}$ [see below for the origin of the critical $R_{\mathrm{c}}$]. A closer look at this evolution of ${\mathscr {D}}(\Delta _{x\mathrm{F}})$ is provided in the inset panel of Fig. 9, in which we label the critical distance $R_{\mathrm{c}}$. Clearly, ${\mathscr {D}}(\Delta _{x\mathrm{F}})$ increases slightly with the gap at certain $R < R_{\mathrm{c}}$, while it increases up to a critical gap at certain $R > R_{\mathrm{c}}$ and starts to decrease. The critical gaps at $R > R_{\mathrm{c}}$ are labeled by the red solid dots. From the contour plot, it is also noteworthy that the size of this critical gap is inversely proportional to the distance *R*, meaning that it decreases with *R*. The rapid change in ${\mathscr {D}}(\Delta _{x\mathrm{F}})$ in terms of the gap edges for $R > R_{\mathrm{c}}$ is the main reason of the suddenly changes in the RKKY response at *R*- and $\varphi _R$-dependent critical gap potentials. The same argument is valid for $\Delta _{y\mathrm{F}}$.

It is worthwhile to understand the reason behind the critical $R_{\mathrm{c}}$ from the susceptibility in Eq. (25). For simplicity, we restrict ourselves to the band edge $\omega = \Delta _{x\mathrm{F}}$ and set $\Delta _{y\mathrm{F}} = 0$. The integrands of the terms show a maximum value of $R_{\mathrm{c}} \simeq 8$ Å, for the considered range of distortion potential $0<\Delta _{x\mathrm{F}}<0.1$, meaning that the slope of this function approaches zero at this critical $R_{\mathrm{c}}$. The same arguments can be set for the case of $\omega = \Delta _{y\mathrm{F}}$ and $\Delta _{x\mathrm{F}} = 0$. It should be restressed that the critical $R_{\mathrm{c}}$ may be different for other directions $\varphi _R$.

**Figure 10:**
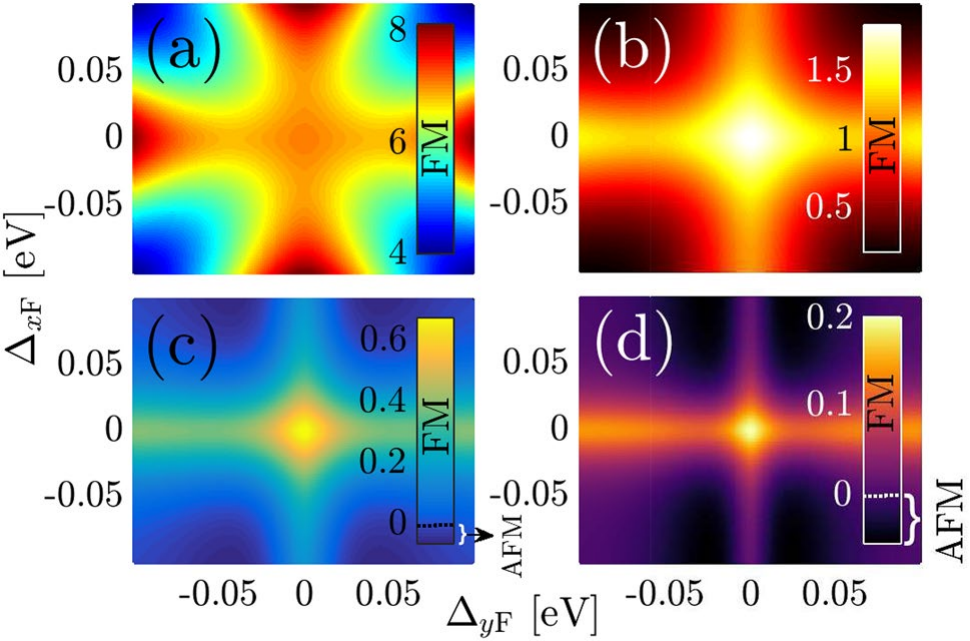
Magnetic phase diagram for the RKKY response ${\mathscr {J}}^{\mathrm{RKKY}}_{\alpha \alpha }(\mathbf{R }, \varphi _R)/ {\mathscr {J}}^2\,C_t \times 10^{-4}$ at $\varphi _R = 0$, to the ferroelectric distortion potentials along both *x*- and *y*-directions at (**a**) $R = 10$ Å, (**b**) $R = 30$ Å, (**c**) $R = 50$ Å, and (**d**) $R = 70$ Å.

To summarize non-trivial behavior of the RKKY interaction in the short and intermediate range of impurity separations, high correlation function at the band edges leads to an enhanced host states in the valence band giving rise to stronger magnetic response. Thereby if magnetic impurities are resided along the gapped Dirac cones, the RKKY interaction would be based on the mentioned mechanism which is increasing with the gap size at short-range distances. It should be reminded that at long-range separations, the RKKY interaction exponentially decays with the gap size. Simultaneously, massless Dirac fermions along the perpendicular direction indirectly affects RKKY response especially when Fermi energy lies in the gap. On the other hand, if impurities are aligned along the gapless Dirac cones, they would couple to each other by means of the massless host electrons while massive Dirac fermions also affects RKKY interaction indirectly via the valence band states [please see Fig. 10d].

For a physical interpretation, we would state that the system possesses a new *quasifermion* in this situations in the presence of simultaneous massless and massive fermions. In fact, one is allowed to define $\psi ^{\mathrm{quasifermion}}(\mathbf{k }) = \sum _{i}{\mathscr {P}}_i \psi ^{\mathrm{massless\,\,fermions}}_i(\mathbf{k }) + {\mathscr {P}}'_i \psi ^{\mathrm{massive\,\,fermions}}_i(\mathbf{k })$ in terms of the orthogonal massless and massive wave functions with corresponding probability distributions $\{{\mathscr {P}}_i,{\mathscr {P}}'_i\}$ for the *i*-th orbital, which are responsible for the nontrivial RKKY treatment compared to the individual massless and massive fermions. We just mention this point here to justify the new created fermions when coexisting gapless and gapped phases. More details of the momentum-dependent Hamiltonian and the band structure of these introduced quasifermions can be tracked from the Eq. (3) and corresponding Fourier transforms, which are out of the scope of the present paper.

#### Anisotropically gapped TCIAnisotropically gapped TCI

In the case of anisotropically gapped TCI, the sign of $\Delta _{x\mathrm{F}}\,\Delta _{y\mathrm{F}}$ is important and one would report the general formulation of the susceptibility in Eq. (25) for this part [see the last row of Fig. 4]. However, the short- and long-range responses can be read as

36a $$\begin{aligned} \frac{\chi ^{\alpha \alpha }_{ii}(\,\mathbf{R }\,,\varphi _R)}{C_t} \propto {}&\frac{{\mathscr {F}}(\mathbf{R }\,,\varphi _R)}{R^3}\, , \end{aligned}$$

36b $$\begin{aligned} \frac{\chi ^{\alpha \alpha }_{ii}(\,\mathbf{R }\,,\varphi _R)}{C_t} \propto {}&\frac{h(\mathbf{R }\,,\varphi _R)}{R^{3/2}}\,, \end{aligned}$$

where $h(\mathbf{R }\,,\varphi _R) = (C_1-1)\,\Delta ^{3/2}_{x\mathrm{F}}\,e^{-2\,\Delta _{x\mathrm{F}}\,R/v_{\mathrm{F}}}+(C_2-1)\,\Delta ^{3/2}_{y\mathrm{F}}\,e^{-2\,\Delta _{y\mathrm{F}}\,R/v_{\mathrm{F}}}+(C_4-C_3)\,\frac{\Delta _{x\mathrm{F}}\,\Delta _{y\mathrm{F}}}{\sqrt{\Delta _{x\mathrm{F}}+\Delta _{y\mathrm{F}}}}e^{-\,\Delta _{x\mathrm{F}}\,R/v_{\mathrm{F}}}\,e^{-\,\Delta _{y\mathrm{F}}\,R/v_{\mathrm{F}}}$. It is not easy to deduce a general treatment for this case due to the complexity of the interference term at long-range impurity separations. But, the decaying rates are similar to two previous cases.

To understand the conditions for which the magnetic phase transition occurs by symmetry breaking and band gap opening in such a situation, in Fig. 10 we show the RKKY interaction strength over the full allowed range of gaps for different values of distance $R > R_{\mathrm{c}}$ between two magnetic impurities with $\varphi _R = 0$. The breakdown of the antiferromagnetic and ferromagnetic phases is governed by the positive and negative signs in the color bars. For a quantitative analysis of ${\mathscr {J}}^{\mathrm{RKKY}}_{\alpha \alpha }(\mathbf{R }, \varphi _R)/ {\mathscr {J}}^2\,C_t$, we label zero response by dotted lines in the color bars. We noted that there is no asymmetric behavior for RKKY response concerning the band inversion effect originating from the gap signs. It should be highlighted that at large separations of Fig. 10d, stronger RKKY coupling is obvious when magnetic moments located along $\varphi _R = 0$ are coupled to each other via the host massless Dirac fermions along the *x*-axis, $\Delta _{x\mathrm{F}}=0$.

In particular, independent of the distance *R*, the amplitude of the RKKY interaction attains a maximum in the absence of any mass term induced by the ferroelectric distortion, while decreases, as discussed before, with the gap potentials. Following the long-range RKKY response in Eq. (36), Fig. 10 shows that for the cases of $\Delta _{x\mathrm{F}}\Delta _{y\mathrm{F}} \ne 0$, the RKKY response decreases exponentially with the gap such that at large-enough distances, the magnetic phase transitions take place. For $R = 10$ Å  [see Fig. 10a] it is no surprise that the ${\mathscr {J}}^{\mathrm{RKKY}}_{\alpha \alpha }(\mathbf{R }, \varphi _R)/ {\mathscr {J}}^2\,C_t$ gets positive values because this is not the effective distance for the sign switching of the RKKY response. For $R = 30$ Å  i.e. Fig. 10b, again, no transition takes place, while for distance $R = 50$ Å  in Fig. 10c, transitions are appearing for $|\Delta _{x/y\mathrm{F}}| \gtrsim 0.06$. However, if we look at the magnetic moments on the same sublattices with longer separation $R= 70$ Å  in Fig. 10d, the transition occurs in a wider range of gaps involving the negative RKKY interaction, namely $|\Delta _{x/y\mathrm{F}}| \gtrsim 0.03$. We comment that for $\varphi _R = \pi /2$, one would find the same feature except that in turn, stronger RKKY coupling would be observed if massless Dirac fermions along the *y*-axis play the role of host carriers, $\Delta _{y\mathrm{F}}=0$.

**Figure 11:**
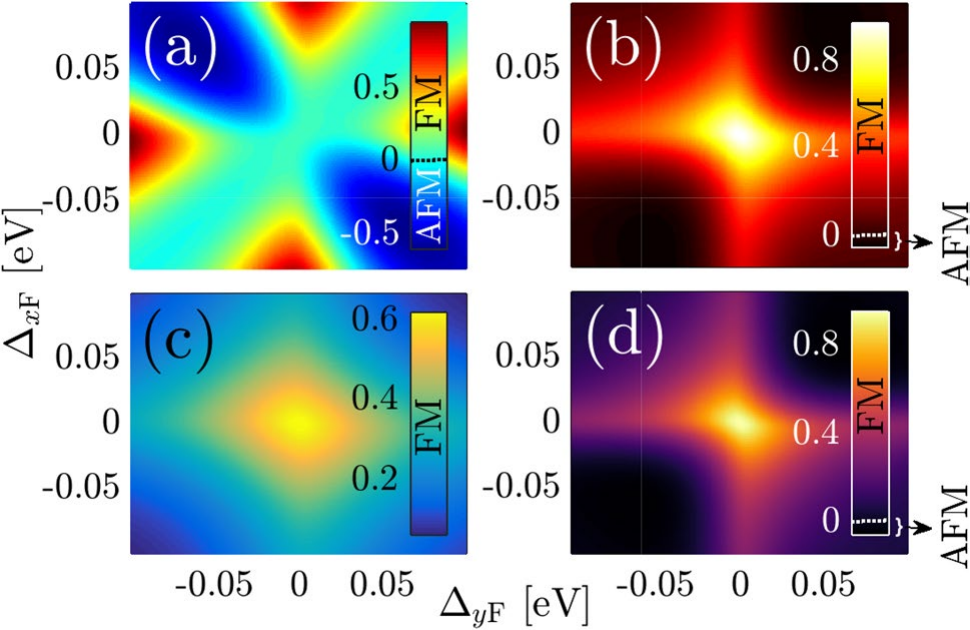
Magnetic phase diagram for the RKKY response ${\mathscr {J}}^{\mathrm{RKKY}}_{\alpha \alpha }(\mathbf{R }, \varphi _R)/ {\mathscr {J}}^2\,C_t \times 10^{-4}$, to the ferroelectric distortion potentials along both *x*- and *y*-directions at (**a**) $\{R = 25\,$Å$,\varphi _R = \pi /6\}$ and (**b**) $\{R = 50\,$Å$,\varphi _R = \pi /6\}$ as well as at (**c**) $\{R = 30\,$Å$,\varphi _R = \pi /4\}$ and (**d**) $\{R = 50\,$Å$,\varphi _R = \pi /4\}$.

For other $\varphi _R$ angels, we discuss RKKY response to the magnetic moments located along the direction $\varphi _R = \pi /6$ for $R = 25$ Å  and 50 Å  in Fig. 11a and b, respectively, as well as with $\varphi _R = \pi /4$ for $R = 30$ Å  and 50 Å  in Fig. 11c and d. For the same range of gaps we examined, the symmetry property of the RKKY interaction with respect to the gap sign is broken, i.e. ${\mathscr {J}}^{\mathrm{RKKY}}_{\alpha \alpha }(\mathbf{R }, \varphi ^{\mathrm{in-plane}}_R, +\Delta _{x/y\mathrm{F}}) \ne {\mathscr {J}}^{\mathrm{RKKY}}_{\alpha \alpha }(\mathbf{R }, \varphi ^{\mathrm{in-plane}}_R, -\Delta _{x/y\mathrm{F}})$. However, regarding the Eq. 25 and numerical results in Fig. 11, the RKKY coupling depends on the sign of $\Delta _{y\mathrm{F}} \Delta _{y\mathrm{F}}$  [see the first and third as well as the second and fourth quarters in all panels of Fig. 11]. The above interpretation is valid for both considered angles.

Regarding the magnetic phase transition at $\varphi _R = \pi /6$ for $R = 25$ Å  for the gaps with the property $\Delta _{x\mathrm{F}}\Delta _{y\mathrm{F}}<0$, the spin flipping occurs, as shown in Fig. 11a. If we seek for such a phase transition at longer distances, however, we find it in the majority of gaps with the property $\Delta _{x\mathrm{F}}\Delta _{y\mathrm{F}}>0$, as shown in Fig. 11b. It is interesting that based on the amplitude variation of the responses, the transition in the short range of $\varphi _R = \pi /6$ is stronger than that of long-range one. In the case of angle $\varphi _R = \pi /4$, the situation is different, and much fewer amplitudes corresponding to the magnetic phase transitions are obtained. While for the short-range RKKY interaction, Fig. 11c, there is no phase transition, a weak transition emerges for the long-range case when the gaps only satisfy the feature $\Delta _{x\mathrm{F}}\Delta _{y\mathrm{F}}>0$.

We notice that the critical susceptibility at which the magnetic phase transition appears depends strongly on the distance *R* and angle $\varphi _R$. Thereby, the behavior of spin susceptibility for two magnetic impurities placed on different atomic sites (which possesses quite different interaction conceptually) may be quite different and notable. In the next section, we will elucidate this.

### Impurities on different sublattices

When two magnetic moments reside on different sublattices of the surface (001) of SnTe, the susceptibility can be calculated as

37 $$\begin{aligned} \chi ^{\alpha \beta }_{ii}(\,\mathbf{R }\,) = \frac{2}{\pi } \int ^{0}_{-\infty } d\omega \,\mathfrak {R}\left[ G^0_{\alpha \beta }(\omega ,\mathbf{R },0)\,G^0_{\alpha \beta }(\omega ,0,\mathbf{R })\right] \, , \end{aligned}$$

resulting in [$$\chi ^{12}_{ii}(\,\mathbf{R }\,,\varphi _R) = \chi ^{21}_{ii}(\,\mathbf{R }\,,\varphi _R)$$]

38 $$\begin{aligned} \begin{aligned} \frac{\chi ^{\alpha \beta }_{ii}(\,\mathbf{R }\,,\varphi _R)}{C_t} = {}&- D_1 \int ^{0}_{-\infty } d\omega \,(\omega ^2+\Delta ^2_{x\mathrm{F}})K^2_1\left( {\mathscr {A}}R/v_{\mathrm{F}}\right) {}-D_2 \int ^{0}_{-\infty } d\omega \, (\omega ^2+\Delta ^2_{y\mathrm{F}})K^2_1\left( {\mathscr {B}}R/v_{\mathrm{F}}\right) \\{}&-D_3 \int ^{0}_{-\infty } d\omega \,{\mathscr {A}}\,{\mathscr {B}}\,K_1\left( {\mathscr {A}}R/v_{\mathrm{F}}\right) \,K_1\left( {\mathscr {B}}R/v_{\mathrm{F}}\right) \, , \end{aligned} \end{aligned}$$

where $$D_1 = 2 \cos ^2(\Lambda _x\,R_x), D_2 = 2 \cos ^2(\Lambda _y\,R_y)$$ and $$D_3 = 4 \cos \gamma \cos (\Lambda _x\,R_x)\cos (\Lambda _y\,R_y)$$.

**Figure 12:**
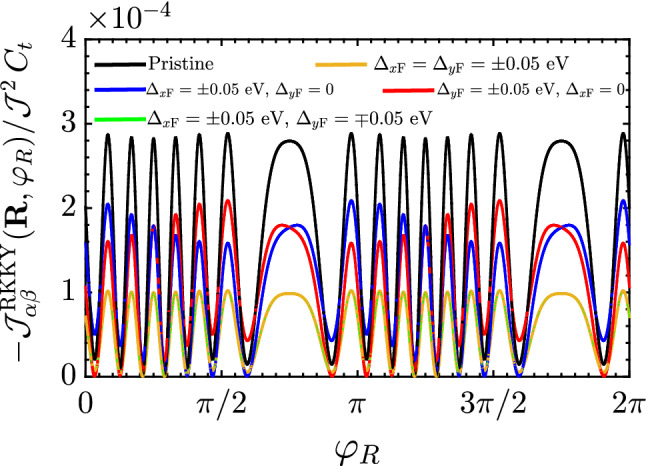
The behavior of the RKKY interaction $-{\mathscr {J}}^{\mathrm{RKKY}}_{\alpha \beta }(\mathbf{R }, \varphi _R)/ {\mathscr {J}}^2\,C_t$ as a function of $\varphi _R$ at fixed $R = 50$ Å  for different ferroelectric distortion potentials.

The emergent conclusions extracting from the above susceptibility, i.e. Eq. (38) could be listed as the following. Firstly, we conclude that the RKKY response is negative anyway, providing an antiferromagnetic spin configuration for impurities sitting on the different sublattices. Secondly, the RKKY interaction is an even function of the gap terms, meaning that the gap term sign is not a matter subject from the point of RKKY response. In other words, it is not possible to detect the magnetic phase transitions when the impurities are placed on different sublattices through the magnetic response, in contrary to the previous the same sublattices case.

**Figure 13:**
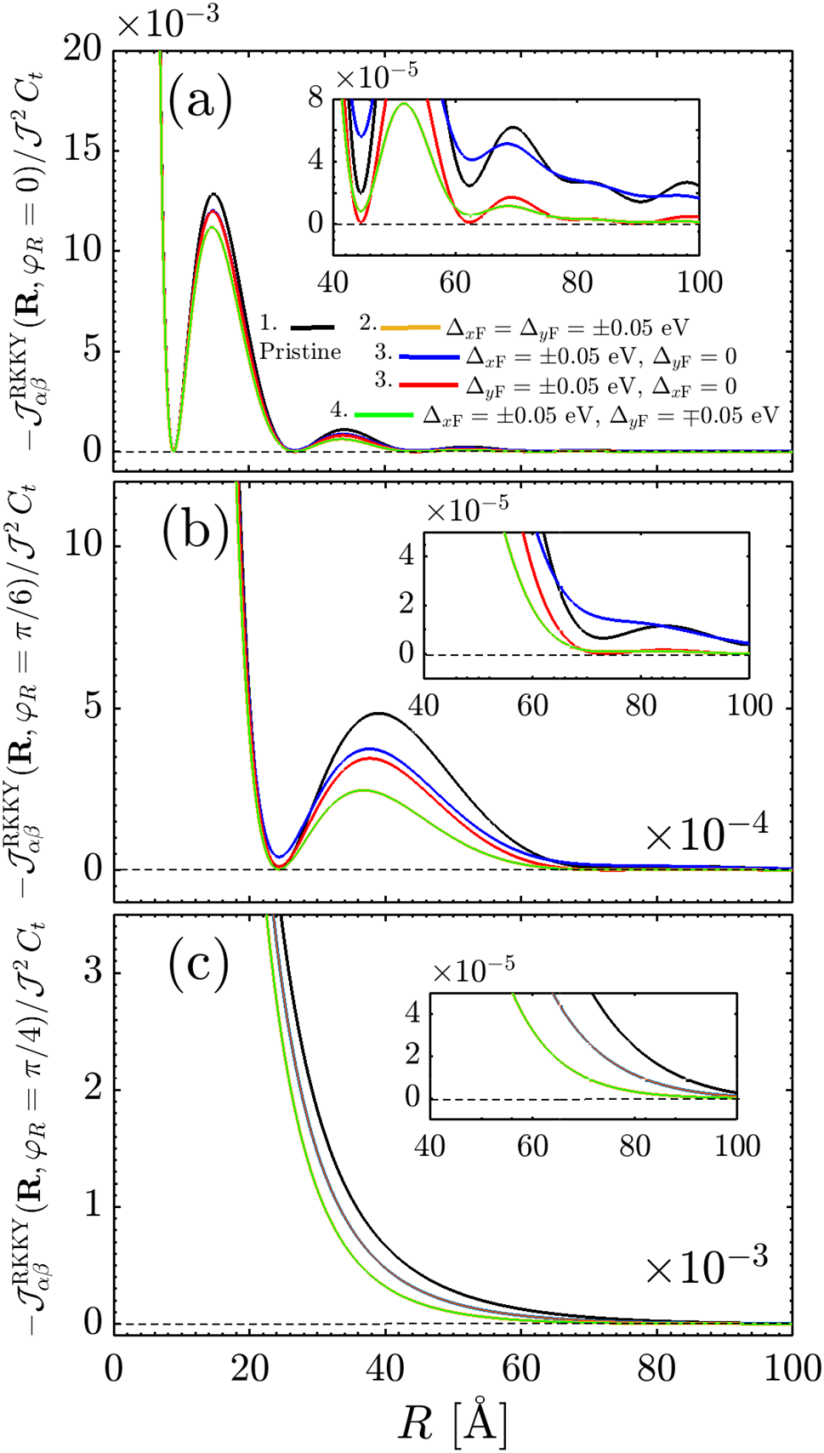
The behavior of the susceptibility $-{\mathscr {J}}^{\mathrm{RKKY}}_{\alpha \beta }(\mathbf{R }, \varphi _R)/ {\mathscr {J}}^2\,C_t$ as a function of *R* for different ferroelectric distortion potentials at (**a**) $\varphi _R=0$, (**b**) $\varphi _R=\pi /6$ and (**c**) $\varphi _R=\pi /4$.

Similar to the same sublattices case, the RKKY response is periodic in $\varphi _R$, as expected from the structure symmetry of the SnTe lattice. This fact is numerically verified in Fig. 12 in the absence and presence of the ferroelectric distortion to highlight the distortion effect on the RKKY intensity. From this plot, one immediately would conclude that the presence of both $\Delta _{x\mathrm{F}}$ and $\Delta _{y\mathrm{F}}$ simultaneously with different possibilities for the sign can not influence the RKKY response [see the orange and green curves on top of each other]. This is a direct consequence of power 2 of the gaps in Eq. (38). Furthermore, it exhibits a beating type pattern with gap-dependent amplitudes originating from the Fermi wave vectors involving in the response.

The RKKY interaction is again strongly anisotropic concerning the direction of applied displacements. However, for a general discussion of the distortion configuration effects on the magnetic response of impurities resided on different sublattices, we again divide the following analyses into four parts considering (1) pristine TCI, (2) isotropically gapped TCI, (3) coexistence of gapless and gapped TCI, and (4) anisotropically gapped TCI.

#### Pristine TCI

Considering $\Delta _{x\mathrm{F}} = \Delta _{y\mathrm{F}} = 0$ [see the first row of Fig. 4] in Eq. (38), one achieves

39 $$\begin{aligned} \begin{aligned} \frac{\chi ^{\alpha \beta }_{ii}(\,\mathbf{R }\,,\varphi _R)}{C_t} \propto {}&-\frac{{\mathscr {L}}(\mathbf{R }\,,\varphi _R)}{R^3}\, , \end{aligned} \end{aligned}$$

where

40 $$\begin{aligned} \begin{aligned} {\mathscr {L}}(\mathbf{R }\,,\varphi _R)={}&D_1 + D_2 + D_3 ={} \cos ^2(\Lambda _x\,R_x)+\cos ^2(\Lambda _y\,R_y){}+2\,\cos \gamma \,\cos (\Lambda _x\,R_x)\,\cos (\Lambda _y\,R_y) ={} {\mathscr {F}}(\mathbf{R }\,,\varphi _R)\, , \end{aligned} \end{aligned}$$

Interestingly, similar interference term as the case of the same sublattices can be obtained, in an excellent agreement with Ref.^43^, while the magnetic ordering is different due to the negative sign of susceptibility referring to the antiferromagnetic phase. It is worth mentioning that the pristine RKKY amplitude is different in this case compared to the same sublattices due to the difference between the modified Bessel functions of the zero and first kinds. The corresponding results are shown as black curves in Fig. 13a–c for which the short- and long-range responses decay as $R^{-3}$ with the impurity separation, whereas RKKY interaction oscillates with intermediate *R* due to oscillating interference term ${\mathscr {L}}(\mathbf{R }\,,\varphi _R)$. However, no magnetic phase transition is observed at all.

#### Isotropically gapped TCI

Turning now to the isotropically gapped TCIs with the feature $|\Delta _{x\mathrm{F}}| = |\Delta _{y\mathrm{F}}| = \Delta _0$ [see the second row of Fig. 4] leads to the expression

41 $$\begin{aligned} \frac{\chi ^{\alpha \beta }_{ii}(\,\mathbf{R }\,,\varphi _R)}{C_t} = {} -2\,{\mathscr {L}}(\mathbf{R }\,,\varphi _R)\,\int ^{0}_{-\infty } d\omega \, \omega ^2\,K^2_1\left( {\mathscr {A}}' R/v_{\mathrm{F}}\right) - {} 2\,{\mathscr {L}}(\mathbf{R }\,,\varphi _R)\,\Delta ^2_0\,\int ^{0}_{-\infty } d\omega \,K^2_1\left( {\mathscr {A}}' R/v_{\mathrm{F}}\right) \, . \end{aligned}$$

With the help of Eqs. (26) and (31), one finds for the short-range $\Delta _0R/v_{\mathrm{F}}\ll 1$

42 $$\begin{aligned} \begin{aligned} \frac{\chi ^{\alpha \beta }_{ii}(\,\mathbf{R }\,,\varphi _R)}{C_t} \propto {} -\frac{{\mathscr {L}}(\mathbf{R }\,,\varphi _R)}{R^3}\, , \end{aligned} \end{aligned}$$

and for the long-range $\Delta _0R/v_{\mathrm{F}}\gg 1$ using the Laplace method

43 $$\begin{aligned} \begin{aligned} \frac{\chi ^{\alpha \beta }_{ii}(\,\mathbf{R }\,,\varphi _R)}{C_t} \propto {} - \frac{{\mathscr {L}}(\mathbf{R }\,,\varphi _R)\,\Delta ^{3/2}_0}{R^{3/2}}\,e^{-2\,\Delta _0\,R/v_{\mathrm{F}}}\, . \end{aligned} \end{aligned}$$

wherein the decaying rates are quite similar to the same sublattices case. However, there is no magnetic phase transition for the short-, intermediate- and long-range impurity separations in contrast to the same sublattices. The reason can be understood from the same interference terms behind the integrals in the equation above, while different interference terms came up in Eqs. (32) and (33). These behaviors are greatly confirmed numerically in orange curves (under the green ones) of the Fig. 13a–c describing the decaying rate above-mentioned for the short- and long-range responses as well as oscillation for the intermediate distances.

**Figure 14:**
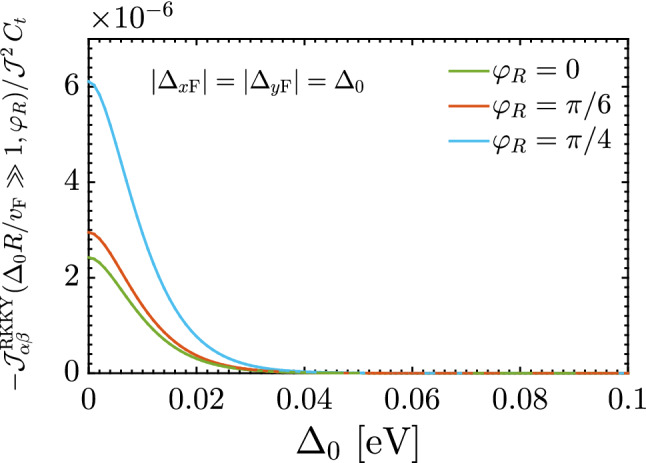
Long-range behavior of the RKKY interaction $-{\mathscr {J}}^{\mathrm{RKKY}}_{\alpha \beta }(\Delta _0R/v_{\mathrm{F}}\gg 1, \varphi _R)/ {\mathscr {J}}^2\,C_t$ as a function of $\Delta _0$ at fixed long-range impurity separation $R = 200$ Å  for different directions $\varphi _R$.

The exponential decaying of the RKKY interaction with the gap potential $-{\mathscr {J}}^{\mathrm{RKKY}}_{\alpha \beta }(\mathbf{R }, \varphi _R)/ {\mathscr {J}}^2\,C_t$ is investigated at long-range regime in Fig. 14 along different directions $\varphi _R$. The process of exponentially decay of the RKKY interaction with the gap potential is the recovery of Eq. (43).

#### Coexistence of gapless and gapped TCI

For the individual case of $|\Delta _{x\mathrm{F}}| = \Delta _0$ and $|\Delta _{y\mathrm{F}}| = 0$ [see the third and fourth rows of Fig. 4], we have

44 $$\begin{aligned} \begin{aligned} \frac{\chi ^{\alpha \beta }_{ii}(\,\mathbf{R }\,,\varphi _R)}{C_t} = {}&-D_1\,\int ^{0}_{-\infty } d\omega \, \omega ^2\,K^2_1\left( {\mathscr {A}}' R/v_{\mathrm{F}}\right) {} -D_1\,\Delta ^2_0\,\int ^{0}_{-\infty } d\omega \,K^2_1\left( {\mathscr {A}}' R/v_{\mathrm{F}}\right) {}-D_2\,\int ^{0}_{-\infty } d\omega \, \omega ^2\,K^2_1\left( \omega R/v_{\mathrm{F}}\right) \\&{} -D_3\,\int ^{0}_{-\infty } d\omega \,\omega \,{\mathscr {A}}'\,K_1\left( \omega R/v_{\mathrm{F}}\right) \,K_1\left( {\mathscr {A}}' R/v_{\mathrm{F}}\right) \, . \end{aligned} \end{aligned}$$

Substituting $D_1 \mapsto D_2$ and $D_2 \mapsto D_1$ in the third first terms give rise to another case of $|\Delta _{y\mathrm{F}}| = \Delta _0$ and $|\Delta _{x\mathrm{F}}| = 0$. Following Eqs. (26) and (31), we obtain respectively the effective short-range $\Delta _0R/v_{\mathrm{F}}\ll 1$ and long-range $\Delta _0R/v_{\mathrm{F}}\gg 1$ responses

45a $$\begin{aligned} \frac{\chi ^{\alpha \beta }_{ii}(\,\mathbf{R }\,,\varphi _R)}{C_t} \propto {}&-\frac{{\mathscr {L}}(\mathbf{R }\,,\varphi _R)}{R^3}\, , \end{aligned}$$

45b $$\begin{aligned} \frac{\chi ^{\alpha \beta }_{ii}(\,\mathbf{R }\,,\varphi _R)}{C_t} \propto {}&-\frac{D_1\,\Delta ^{3/2}_0}{R^{3/2}}\,e^{-2\,\Delta _0\,R/v_{\mathrm{F}}}\, , \end{aligned}$$

showing the same decaying rates as the previous case, while different interference terms emerge. Also, a simple comparison between blue and red curves in Fig. 13 and Eq. (45) alarms that the short-range RKKY responses are somewhat gap-independent, whereas the long-range response comes up with a difference in oscillations for different configurations. It is necessary to mention that the decaying rates here also behave similarly to graphene with oscillations depending on the various gap configurations^49–51^.

As expected, the difference between the case of $\{|\Delta _{x\mathrm{F}}| = \Delta _0,|\Delta _{y\mathrm{F}}| = 0\}$ and $\{|\Delta _{y\mathrm{F}}| = \Delta _0,|\Delta _{x\mathrm{F}}| = 0\}$ belong to the long-range responses except the case of $\varphi _R = \pi /4$ [Fig. 13c] for which both behave similarly independent of the impurity separation *R* because in this case, we have $D_1 = D_2$ and there is no priority for these configurations at all.

**Figure 15:**
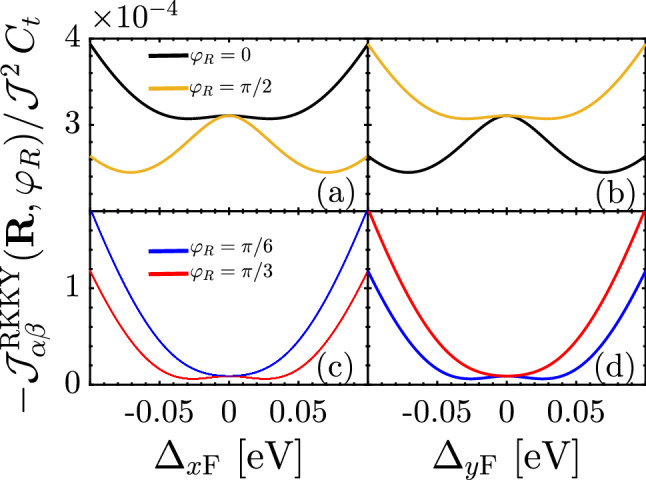
The behavior of the RKKY interaction $-{\mathscr {J}}^{\mathrm{RKKY}}_{\alpha \beta }(\mathbf{R }, \varphi _R)/ {\mathscr {J}}^2\,C_t$ as a function of ferroelectric distortion potential (**a**), (**c**) $\Delta _{x\mathrm{F}}$ and (**b**), (**d**) $\Delta _{y\mathrm{F}}$ for four different complementary angels $\varphi _R$ between two magnetic moments at $R = 25$ Å.

The effect of gap formation on the RKKY interaction at an intermediate impurity separation of $R = 25$ Å  is studied in Fig. 15 for the complementary angles $\varphi _R$ when the distortion is applied along the $X_1-\Gamma -X_1$ line or along its perpendicular orientation $X_2-\Gamma -X_2$. The response is monotonically antiferromagnetic for all gap terms, as expected. Moreover, a correspondence between the distortion direction and the substitutional orientation of impurities is observed in Fig. 15, i.e. the RKKY response for the distortion applied along the *y*-axis with sitting impurities along $\varphi _R=0$ corresponds to the situation in which the distortion is applied along the *x*-axis with $\varphi _R=\pi /2$. Again we observe an increased treatment of the RKKY interaction after a critical gap, which is originated from the high density of the band edges in short-range distances or the role of metallic states along the other direction. The physic behind this enhancement is similar to what we presented for the same sublattices.

We comment that this is also a nontrivial phenomenon as the same sublattices case when both gapless and gapped phases coexist in the whole band spectrum. An increase behavior of the RKKY coupling with the gap size at intermediate-range of impurity separations is traced back to those accumulated valence band states at the band edges affecting RKKY response. Interestingly, such nontrivial increasing trend occurs if magnetic impurities are located along arbitrary orientations. Let us turn to the last distortion configuration.

#### Anisotropically gapped TCI

Referring again to Eq. (38), the situation of interest for the presence of both $\Delta _{x\mathrm{F}}$ and $\Delta _{y\mathrm{F}}$ is when they compete to change the RKKY amplitude [see the last row of Fig. 4], in which the effective short-range $\Delta _0R/v_{\mathrm{F}}\ll 1$ and long-range $\Delta _0R/v_{\mathrm{F}}\gg 1$ responses are given by

46a $$\begin{aligned} \frac{\chi ^{\alpha \beta }_{ii}(\,\mathbf{R }\,,\varphi _R)}{C_t} \propto {}&-\frac{{\mathscr {L}}(\mathbf{R }\,,\varphi _R)}{R^3}\, , \end{aligned}$$

46b $$\begin{aligned} \frac{\chi ^{\alpha \beta }_{ii}(\,\mathbf{R }\,,\varphi _R)}{C_t} \propto {}&-\frac{{\mathscr {M}}(\mathbf{R }\,,\varphi _R)}{R^{3/2}}\, , \end{aligned}$$

where

47 $$\begin{aligned} {\mathscr {M}}(\mathbf{R }\,,\varphi _R) = {} D_1\,\Delta ^{3/2}_{x\mathrm{F}}\,e^{-\frac{2\,\Delta _{x\mathrm{F}}\,R}{v_{\mathrm{F}}}} + D_2\,\Delta ^{3/2}_{y\mathrm{F}}\,e^{-\frac{2\,\Delta _{y\mathrm{F}}\,R}{v_{\mathrm{F}}}} {} + D_3\,\frac{\Delta _{x\mathrm{F}}\,\Delta _{y\mathrm{F}}}{\sqrt{\Delta _{x\mathrm{F}}+\Delta _{y\mathrm{F}}}}\,e^{-\frac{\,\Delta _{x\mathrm{F}}\,R}{v_{\mathrm{F}}}}\,e^{-\frac{\,\Delta _{y\mathrm{F}}\,R}{v_{\mathrm{F}}}}\, . \end{aligned}$$

**Figure 16:**
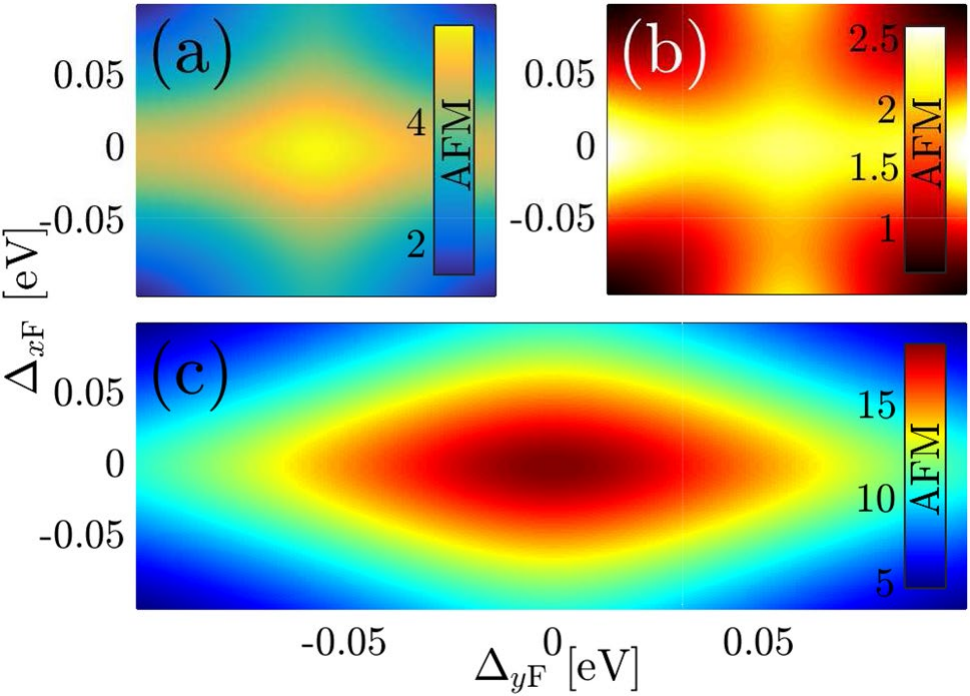
Magnetic phase diagram for the RKKY response ${\mathscr {J}}^{\mathrm{RKKY}}_{\alpha \beta }(\mathbf{R }, \varphi _R)/ {\mathscr {J}}^2\,C_t \times 10^{-4}$ at $R = 30$ Å , to the ferroelectric distortion potentials along both *x*- and *y*-directions at (**a**) $\varphi _R = 0$, (**b**) $\varphi _R = \pi /6$ and (**c**) $\varphi _R = \pi /4$.

We again intend to systematically study the impact of the competition between $\Delta _{x\mathrm{F}}$ and $\Delta _{y\mathrm{F}}$ on the RKKY response. To do so, we plot Fig. 16 at an intermediate distance $R = 30$ Å  for different directions, namely (a) $\varphi _R = 0$, (b) $\varphi _R = \pi /6$ and (c) $\varphi _R = \pi /4$. Independent of the direction $\varphi _R$, the RKKY response is symmetric concerning $\Delta _{x\mathrm{F}}\,\Delta _{y\mathrm{F}} < 0$ or $\Delta _{x\mathrm{F}}\,\Delta _{y\mathrm{F}} > 0$, in which the RKKY amplitude is weaker than the other areas. Moreover, we once more confirm that there is no magnetic phase transition at all when the magnetic impurities are resided on different sublattices. A quick comparison between different directions shows that the case of $\varphi _R = \pi /4$ leads to the maximum RKKY amplitude due to the equal contribution of the Fermi wave vectors in $D_i$ coefficients.

Regarding different magnetic ordering of two configurations (when two impurities residing on the same or different sublattices), let us briefly mention that atomic orbitals and topology of the structure play the main role in magnetic ordering. Hamiltonian in Eq. (5) is written in the basis set of Te and Sn *p*-orbitals. When the same sublattices are considered, the packing factor is lower compared to the different sublattice case. It means that atomic separation in the same sublattice case is larger than the different sublattice case. Therefore, it is reasonable if the overlapping of the *p*-orbitals between neighbors atoms influences RKKY coupling. The effect of the atomic structure on RKKY interaction is seen also in graphene such that magnetic ordering along direction AA-zigzag is ferromagnetic while it is antiferromagnetic for AB-zigzag direction^48–51^.

### Discussion

The current distortion-induced RKKY response for the magnetic impurities on the same and different sublattices are for the case of undoped Dirac cones. However, we should mention that the presence of electron and/or hole-doping changes the results and the competition between the distortion and doping concentration is an important study because doping may induce new magnetic phases to the system due to the breakdown of the lattice symmetry^43,48,51^.

Additionally, surface roughness as a perturbation preserving time-reversal symmetry is always present in real materials. This perturbation breaks the mirror symmetry locally on the surface, however, the mirror symmetry may still be preserved on the average macroscopically point of view, i.e. when the variation of the atomic places is slow enough^7^. Thereby, we argue that, in this case, the gapless results work out well at long-range impurity separations, while one needs to apply a continuous gapped Dirac model for short-range distances on average. Thus, in the smooth variation of roughness for short distances, the gap is opened in the band spectrum, and so our results for RKKY interaction at short ranges can be considered, while for long-range distances the gapless results are applicable. In the presence of sharp variation of the atomic positions, one should similarly look at the mirror symmetry whether it is preserved or not.

Furthermore, we would state that the current results are based on the zero temperature, however; the temperature dependency of short- and long-range magnetic couplings in the absence and presence of symmetry breaking and doping can be studied as well within the finite-temperature self-consistent field approximation^58,59^. Temperature highlights the screening response of the conducting electrons, which strongly affects the indirect exchange interaction between magnetic impurities, resulting in temperature-dependent magnetic phase transitions.

Finally, we emphasize that the magnetic scattering potential may also happen in the presence of magnetic impurities. In such a situation, the RKKY interaction should be approached beyond the linear response theory and the local magnetization should also be considered^60,61^. This revises the theory of linear off-resonant RKKY interaction between magnetic impurities and considers the impact of impurity resonances. We leave the results of all these matters to come up in our future works.

## Summary

In summary, we have addressed the ferroelectric distortion effects on the RKKY interaction between two magnetic impurities on the (001) surface of SnTe and related alloys as available TCIs. We have shown that the low-energy Hamiltonian of TCIs receives a mirror symmetry breaking between multiple Dirac cones in the presence of the distortion, ensuring the gap at Dirac points. The coexistence of the gapless and gapped phases as well as the isotropically and anisotropically gapped phases grant a fascinating interference correlation between magnetic moments, which manifest themselves in different magnetic ordering. While other Dirac materials can be gapped or gapless, the key point of the present paper, especially is the *the coexistence of the gapless and gapped phases* and their *nontrivial and novel* fingerprints in the *gap-induced RKKY response*. The results are significantly different compared to the pristine TCI and other Dirac materials.

Our numerical results present different magnetic phase diagrams depending on the position of impurity magnetic moments on the (001) surface of TCIs. We have proposed a critical impurity separation of $R_{\mathrm{c}}$ for which the RKKY coupling shows different behaviors with the distortion strength for separations beneath and above $R_{\mathrm{c}}$. Although the distortion does not lead to a magnetic phase transition for the magnetic moments resided on different sublattices, an irregular ferromagnetic $\leftrightarrow$ antiferromagnetic ordering emerges as soon as the impurities reside on the same sublattices with the separation above $R_{\mathrm{c}}$. We further demonstrated that it is feasible to manipulate this magnetic ordering by tuning the distortion strength. Our work paves the way to design protocols in tuning the multiple surface states in TCIs using the external magnetic impurities and ferroelectric distortion for the spintronic and valleytronic applications.

## Data Availability

The data that support the findings of this study are available from the corresponding author upon reasonable request.
